# Shu: visualization of high-dimensional biological pathways

**DOI:** 10.1093/bioinformatics/btae140

**Published:** 2024-03-07

**Authors:** Jorge Carrasco Muriel, Nicholas Cowie, Shannara Taylor Parkins, Marjan Mansouvar, Teddy Groves, Lars Keld Nielsen

**Affiliations:** The Novo Nordisk Foundation Center for Biosustainability, Technical University of Denmark, DK-2800 Kgs. Lyngby, Denmark; The Novo Nordisk Foundation Center for Biosustainability, Technical University of Denmark, DK-2800 Kgs. Lyngby, Denmark; The Novo Nordisk Foundation Center for Biosustainability, Technical University of Denmark, DK-2800 Kgs. Lyngby, Denmark; Department of Biotechnology and Biomedicine, Technical University of Denmark, DK-2800 Kgs. Lyngby, Denmark; The Novo Nordisk Foundation Center for Biosustainability, Technical University of Denmark, DK-2800 Kgs. Lyngby, Denmark; Australian Institute for Bioengineering and Nanotechnology (AIBN), The University of Queensland, St Lucia, QLD 4067, Australia

## Abstract

**Summary:**

Shu is a visualization tool that integrates diverse data types into a metabolic map, with a focus on supporting multiple conditions and visualizing distributions. The goal is to provide a unified platform for handling the growing volume of multi-omics data, leveraging the metabolic maps developed by the metabolic modeling community. In addition, shu offers a streamlined python API, based on the Grammar of Graphics, for easy integration with data pipelines.

**Availability and implementation:**

Freely available at https://github.com/biosustain/shu under MIT/Apache 2.0 license. Binaries are available in the release page of the repository and the web application is deployed at https://biosustain.github.io/shu.

## 1 Introduction

Systems biology produces large amounts of data of varying nature across different experimental conditions (Cvijovic *et al.* 2014). By including multiple layers of information (RNA levels, protein concentrations, metabolite abundances, etc.), multi-omics experiments promise to generate a more holistic view of biological processes. However, even when models are available that can extract the information in multi-omics datasets, it remains a challenge to represent such complex information visually (Agamah *et al.* 2022). Our application shu aims to address this challenge by providing a framework for adding complex visualizations to metabolic maps.

Metabolic maps are graphs where nodes are metabolites and edges are the reactions connecting them. For metabolic engineers, these maps are a valuable tool to contextualize biological data in a representation that they are very familiar with. In the field of genome-scale metabolic modeling, the metabolic maps provided by Escher (King *et al.* 2015) have been applied highly successfully to depict the results of metabolic engineering (Masson *et al.* 2023). Other software tools have built on or followed Escher with further metabolic applications like Caffeine (Lopez *et al.* 2019), CNApy (Thiele *et al.* 2022), Omix (Droste *et al.* 2013), Cytoscape (Shannon *et al.* 2003) or Pathway tools (Karp *et al.* 2021), and many others (Copeland *et al.* 2012).

We aimed to address two outstanding problems with current metabolic network visualization tools. First, the existence of distributional data in the context of a metabolic map. Existing metabolic maps rely on continuous color scales or sizes which can only represent a single value per reaction or metabolite. Often, a full distribution is necessary to avoid losing important information about the modeled behavior. This is even more significant when a central estimate of the distribution is not fully informative, such as when kurtosis is of interest or for skewed or multi-modal distributions. This poses a key challenge when dealing with high-throughput technologies and especially with the output of computational biology models that provide a distribution as a result (such as Medlock *et al.* 2020, Matos *et al.* 2022).

The second problem we address is the need to compare data from multi-omics datasets between experimental conditions without changing the metabolic map. For example, differential omics experiments are very common in biology (Monti *et al.* 2019) and their insights are naturally brought to light only when two or more conditions are visualized together.

Our application shu: a software application that can be used both natively and in a web application to visualize different kinds of information on top of metabolic maps, with emphasis on the representation of distributions and different conditions. In addition, we developed a Python package to enable programmatic access to shu.

## 2 Availability

Documentation for the usage of shu and ggshu is available at https://biosustain.github.io/shu/docs. The Rust API is documented at https://docs.rs/shu. Binaries for Linux, Mac and Windows are distributed for every version and a WebAssembly application was compiled and deployed to be used directly in the browser at https://biosustain.github.io/shu. Data, models and maps are available at https://github.com/biosustain/shu_case_studies.

Given the possible complexity of the data that shu visualizes, a Python package called ggshu is made available to enable users to render this data programmatically, generating the desired JSON file to plot in the metabolic map.

## 3 Materials and methods

Shu is written in the Rust Programming Language (Matsakis and Klock 2014) on top of the open-source bevy Entity-Component-System framework (https://github.com/bevyengine/bevy/releases/tag/v0.11.3).

Shu requires two inputs: the metabolic map and the biological data. The metabolic map works seamlessly with the Escher JSON format. This compatibility enables the use of existing Escher maps, which have been created by the metabolic engineering community to visualize a wide range of organisms and metabolic pathways.

Biological data (also a JSON, ending with “metabolism.json”) is mapped to the identifiers of metabolites, reactions, and conditions. It is represented by three kinds of geometric entities on the map: edges and nodes with configurable colors and sizes; boxes at each side of the edge; and distribution plots including histograms and smooth kernel density plots. As in Escher and other metabolic maps, colors and/or sizes of edges and nodes, can represent a continuous 1D variable per reaction and metabolite.

Box points in shu refer to colored squares that map a continuous variable to a continuous color scale associated with a reaction. Unlike edge properties, box point colors enable us to represent multiple conditions simultaneously by vertical stacking.

Uniquely in shu, histograms and kernel density curves can be plotted alongside edges and nodes to map distributional variables. For edges, three positions can be used to plot three different variables: the left side, the right side and the hovering window. The axis scale is consistent across all plots, allowing for easy comparison of the same variable across different reactions. Since the placement heuristics might fail for complex reactions, the axis can be moved, rotated and scaled freely using the mouse. The positions of the axis can be saved to a new map via the Graphical User Interface.

One or all conditions can be selected to be displayed in the map. When all conditions are selected, the distribution plots and box points can be compared next to each other. Conveniently, a legend is automatically generated only for the geometrical entities associated with data and the selected conditions.

## 4 Case studies

Two case studies are presented to highlight the usefulness of shu for visualizing the output of computational models and experimental multi-omics data. A jupyter notebook for each case study is available in the Supplementary Material for the data integration with and without the python package.

### 4.1 Visualization of distributions from a Bayesian kinetic model across N-conditions

A small kinetic model (23 reactions, 27 metabolites, and 24 enzymes) of glycolysis and the pentose phosphate pathway of *Escherichia coli* was fitted using Maud, a computational Bayesian modeling software for fitting kinetic models (https://github.com/biosustain/Maud). Experimental data were simulated assuming that the means of the prior distributions were correct (see github for details).

**Figure 1.**
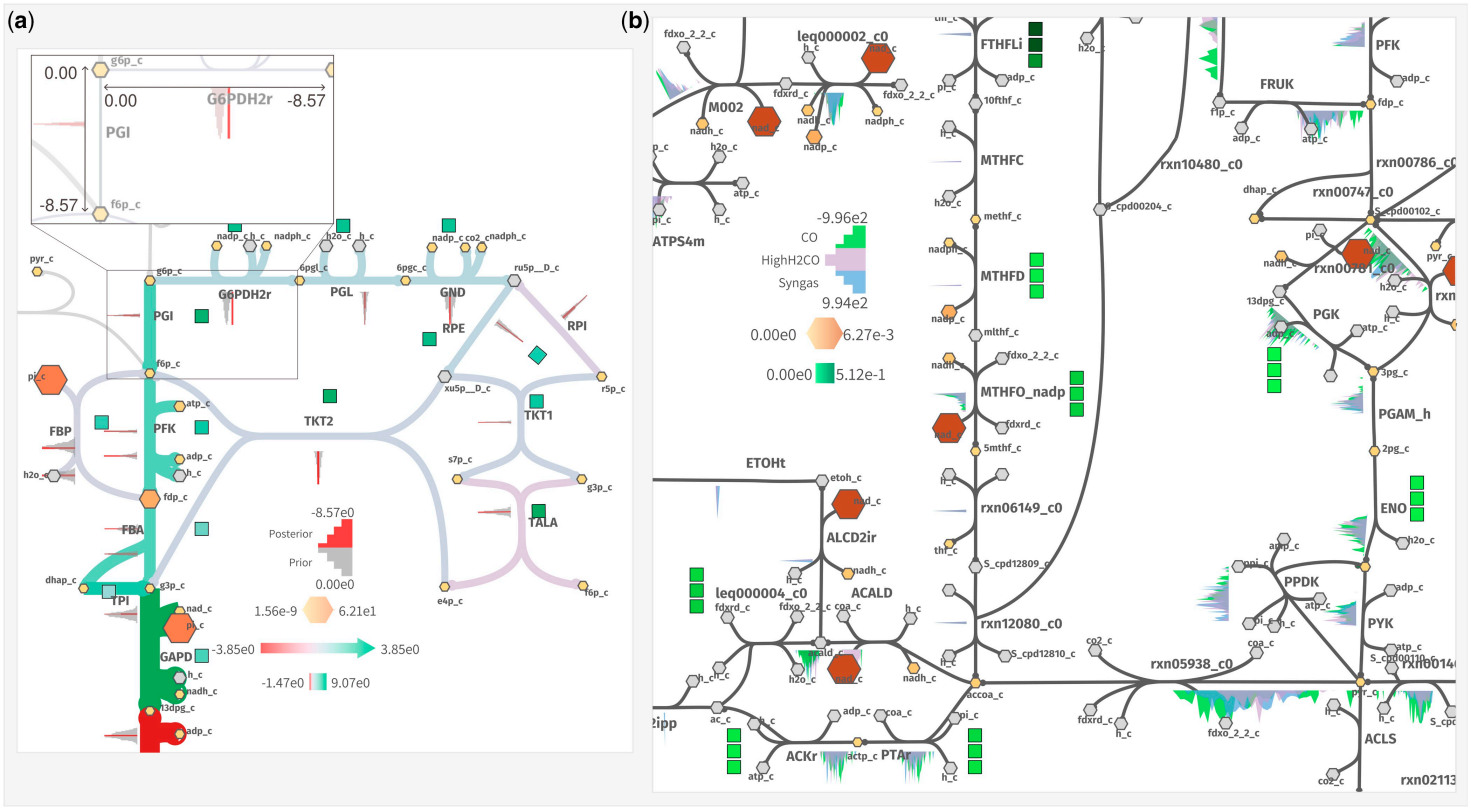
(a) Kinetic Bayesian model displayed in shu: posterior concentrations as sizes and colors of metabolites, log-k_cat_ values as box color and flux as color and size of edges in absolute scale. Priors and posteriors of enzyme log-concentrations are shown as histograms. The axes are annotated in an overlay for reactions PGI and G6PDH2r. (b) Metabolomics, proteomics, and flux sampling plotted as the size and color of metabolites; the color of the boxes; and the density curves respectively. Box conditions top to bottom: CO, H2-High CO, and Syngas.

Figure 1a shows how shu can be used to display posterior draws from the Bayesian metabolic model. In particular, *k*_cat_ constants, enzyme concentrations, fluxes and metabolite concentrations were selected for visualization. Figure 1a shows how these values were mapped with the “Settings” window for reference. The overlay in Fig. 1a shows how the x-limits displayed in the legend are shared across reactions to facilitate comparisons between the enyzme log-concentrations represented by histograms. Hence, it is easy to make assertions such as “GAPD (bottom left) has the highest enzyme concentration in this model.”

### 4.2 Proteomics, metabolomics, and computational fluxes of *Clostridium autoethanogenum*

Different experimental conditions of metabolomics, proteomics and computational fluxes for *Clostridium autoethanogenum* were gathered from de Souza Pinto Lemgruber *et al.* (2018) and Valgepea *et al.* (2018, 2022). This facultative chemolithotroph can grow under varying conditions of CO_2_, CO and H_2_ producing different byproducts of varying degrees of interest for industrial production: acetate, ethanol, 2,3-butanediol, etc. The proteomics and metabolomics datasets were collected for three conditions under different H_2_/CO_2_/CO gas mixtures: “syngas,” “high-H2 CO,” and “CO” in the high biomass setup (1.4 gDCW/L), as explained in the original experimental setup (Valgepea *et al.* 2018). The genome-scale metabolic model of *C.autoethanogenum* iCLAU786 (Valgepea *et al.* 2017) was used to generate flux distributions for the three different conditions by constraining the exchange fluxes of the byproducts and sampling distributions using optGp (Megchelenbrink *et al.* 2014) method in COBRApy (Ebrahim *et al.* 2013). These were mapped to densities in the map.

The result is shown in Fig. 1b. The enzyme concentrations were depicted in the left boxes while the metabolite concentrations were mapped as the color and size of the nodes. The map allows for both global and local observations. Globally, looking at the distribution of fluxes reveal that the Wood–Ljungdahl pathway (*FTHFLi* to *rnx12080_c0*) is the most constrained pathway in the simulation (short peaks of data) while the rest of reactions span across several degrees of magnitude. At the same time, locally, individual reactions can be inspected to make observations about, for instance, the irreversibility of a reaction, like ENO or ACKr. Also, green boxpoints make it easier to see how the most varying concentration of an enzyme is FTHFLi, while the remaining enzymes have similar values across conditions.

## 5 Conclusion

Shu enables the visualization of distributed or 1D data from integrated multi-omics experiments that span multiple experimental conditions. This is particularly useful for histograms, box points, and density functions in publication-ready figures that can simultaneously display all conditions. Since edge colors can only display one condition at a time, reaction data in different conditions can be mapped to box points instead, sorted vertically to display all point estimates across all conditions. Thus, a single figure can be used to represent different conditions juxtaposed to each other without having to rely on more than one visualization of the same map side by side.

As a final remark, we emphasize maps of metabolism because of the prior success in this area coupled with the ready availability of metabolic maps. Shu is however not tied to metabolism and could be used for other kinds of networks, which are a pervasive representation in biology and beyond.

## Supplementary Material

btae140_Supplementary_Data
