# Supplementary material for: Shu: visualization of high-dimensional biological pathways
Source: Bioinformatics. 2024 Mar 7;40(3):btae140. doi: 10.1093/bioinformatics/btae140 (PMC10957514; doi:10.1093/bioinformatics/btae140)
Supplement: btae140_Supplementary_Data [file btae140_supplementary_data.zip › shu_case_studies/models/maud_ecoli_glycolysis_and_ppp/Enzymes for KM (1).pdf]

Transport - PTS

Reaction equation

$1\ PEP + 1\ GLC\ \rightarrow 1\ G6P + 1\ PYR$

phosphoenolpyruvate + glucose = glucose-6-phophate + pyruvate

BIGG: -1 pep_c -1 glc__D_p +1 g6p_c +1 pyr_c

Enzymes and genes

EcoCyc and UniProt

PTS (phosphotransferase system) encoded by

Regulation

Genetic regulation:

Activators:

Inhibitors:

Reaction and enzyme constants

**Parameter**	**Value**	**Uncertainty**	**Reference**
Reversible			
k_{cat}			
Formation energy X			
K_M			
K_I			

Glycolysis - PGI

Reaction equation

$1\ G6P \rightarrow 1\ F6P$

glucose 6-phosphate = fructose 6-phosphate

BIGG: -1 g6p_c +1 f6p_c (glycolytic direction)

Enzymes and genes

EcoCyc and UniProt

PGI (glucose 6-phosphate isomerase or phosphoglucose isomerase) is a homodimeric enzyme encoded by the *pgi* gene and located in the cytosol. A Δ*pgi* strain is viable, but grows slower on glucose since the glucose is used via pentose phosphate pathway causing overproduction of NADPH and the Glx shunt is active in this strain.

Regulation

Gene regulation: SoxS (DNA-binding transcription factor) activates transcription initiation of *pgi*.

Activators: Activation by CrsA (carbon storage regulator; Sabnis et al., 1995).

Inhibitors: Competitive inhibition by PEP to F6P (phosphoenolpyruvate; Ogawa et al., 2007), (allosteric?) inhibition by 6PGC which is part of pentose phosphate pathway (6-phosphogluconate; Schreyer and Böck, 1980) and inhibition by S6P (sorbitol 6-phosphate; Friedberg, 1972).

Reaction and enzyme constants

Parameter	Value	Uncertainty	Reference	In current model
Reversible	yes	-	Ishii et al., 2007	-
k_{cat}	1550.0 1/s	0.2 (chosen)	Ishii et al., 2007 (calculated from specific activity)	yes
k_{cat}	120.5 1/s	-	Gao et al., 2005 (yeast)	no
Formation energy G6P	-1304.7 kJ/mol	1.3 kJ/mol	eQuilibrator	yes
Formation energy F6P	-1302.1 kJ/mol	1.3 kJ/mol	eQuilibrator	yes
K_M G6P	3.0 mM	0.2 (chosen)	Ishii et al., 2007	yes
K_M G6P	1.018 mM	0.142 mM	Ogawa et al., 2007	no
K_M G6P	0.28 mM	-	Gao et al., 2005 (yeast)	no
K_M F6P	0.16 mM	0.2 (chosen)	Ishii et al., 2007	yes
K_M F6P	0.078 mM	0.009 mM	Ogawa et al., 2007	no
K_M F6P	0.2 mM	-	Schreyer and Böck, 1980	no
K_M F6P	0.147 mM	0.006 mM	Gao et al., 2005 (yeast)	no
K_I PEP	0.26 mM	0.5 (chosen)	Ogawa et al., 2007	no
K_I 6PGC	0.19 mM	0.5 (chosen)	Schreyer and Böck, 1980	no

Glycolysis - PFK

Reaction equation

$1\ F6P + 1\ ATP \rightarrow 1\ FDP + 1\ ADP$

fructose 6-phosphate + ATP = fructose 1,6-bisphosphate + ADP

BIGG: -1 f6p_c -1 atp_c +1 fdp_c +1 adp_c (glycolytic direction)

Enzymes and genes

EcoCyc PfkA, EcoCyc PfkB and UniProt PfkA, UniProt PfkB

The PFK (phosphofructokinase) reaction consists of two isozymes: PfkA (PFK I) encoded by the *pfkA* gene and PfkB (PFK II) encoded by the *pfkB* gene. Both isozymes are present in the cytosol, however more than 90% of the total reaction activity in the wild type can be attributed to PfkA (Kotlarz et al., 1975). PfkA is a homotetrameric enzyme, while PfkB is a homodimeric enzyme. A Δ*pfkA* strain is viable and found to increase the NADPH production via the pentose phosphate pathway, while a Δ*pfkB* strain shows no apparent effect. Mg^{2+} is a cofactor of both PfkA and PfkB. Kotlarz and Buc (1982) has a nice overview of kinetics and allosteric regulations of both PfkA and pfkB!

Regulation

Gene regulation: F6P (fructose 6-phosphate) binds to Cra (DNA-binding transcription factor) to block inhibition, while Cra inhibits transcription initiation of *pfkA*, thus an increase in F6P increases transcription of *pfkA*. Nac (DNA-binding transcription factor) activates transcription initiation of *pfkB*, while L-arginine binds to ArgR (DNA-binding transcription factor) to enable inhibition and ArgR inhibits transcription initiation of *pfkB*.

Activators: Allosteric activation of PfkA by ADP (also GDP; Blangy and Monod, 1968) and by F6P (Johnson and Reinhart, 1992), and (product) activation of PfkA by FDP (fructose 1,6-bisphosphate) (Blangy and Monod, 1968). Allosteric activation of PfkB by HPr (phosphocarrier protein; Rodionova et al., 2017).

Inhibitors: Allosteric inhibition of PfkA by PEP (phosphoenolpyruvate) and by ATP (Blangy and Monod, 1968; Berger and Evans, 1991). Allosteric inhibition of PfkB by ATP (Kotlarz et al., 1975; Kotlarz and Buc, 1981), competitive inhibition of PfkB by FDP (fructose 1,6-biphosphate; Campos et al., 1984), and (allosteric) inhibition of PfkB by Pi (phosphate; Parducci et al., 2006).

Reaction and enzyme constants

Parameter	Value	Uncertainty	Reference	In current model
Reversible	no	-	-	-
k_{cat} PfkA	110.0 1/s	0.2 (chosen)	Blangy and Monod, 1968	no
k_{cat} PfkA	130.0 1/s	0.2 (chosen)	Berger and Evans, 1991	yes
k_{cat} PfkA	27.4 1/s	-	Ogawa et al., 2007 (calculated from specific activity)	no
k_{cat} PfkA	120.0 1/s	-	Auzat et al., 1994	no
k_{cat} PfkA	93.0 1/s	-	Hellinga and Evans, 1987	no
k_{cat} PfkA	88.0 1/s	-	Wang and Kemp, 2001	no
k_{cat} PfkA	167.0 1/s	-	Zheng and Kemp, 1995	no
k_{cat} PfkB	53.0 1/s	0.2 (chosen)	Parducci et al., 2006	yes
k_{cat} PfkB	56.0 1/s	0.2 (chosen)	Villalobos et al., 2016	no
k_{cat} PfkB	61.0 1/s	8 1/s	Baez et al., 2013	no
Formation energy F6P	-1302.1 kJ/mol	1.3 kJ/mol	eQuilibrator	yes
Formation energy FDP	-2194.8 kJ/mol	2.1 kJ/mol	eQuilibrator	yes
Formation energy ATP	-2280.7 kJ/mol	2.9 kJ/mol	eQuilibrator	yes
Formation energy ADP	-1405.9 kJ/mol	2.4 kJ/mol	eQuilibrator	yes
K_M F6P of PfkA	0.0125 mM	0.2 (chosen)	Blangy and Monod, 1968	yes
K_M F6P of PfkA	0.030 mM	-	Berger and Evans, 1991	no
K_M F6P of PfkA	0.45 mM	-	Ogawa et al., 2007	no
K_M F6P of PfkA	0.34 mM	-	Auzat et al., 1994	no
K_M F6P of PfkA	0.038 mM	-	Hellinga and Evans, 1987	no
K_M F6P of PfkA	0.16 mM	-	Zheng and Kemp, 1995	no
K_M FDP of PfkA	3.5 mM	0.2 (chosen)	Babul, 1978	yes
K_M FDP of PfkA	15 mM	1.5 (chosen)	Denis' model or unknown	no
K_M ATP of PfkA	0.06 mM	0.2 (chosen)	Blangy and Monod, 1968	yes
K_M ATP of PfkA	0.06 mM	-	Berger and Evans, 1991	no
K_M ATP of PfkA	0.018 mM	-	Ogawa et al., 2007	no
K_M ATP of PfkA	0.057 mM	-	Auzat et al., 1994	no
K_M ATP of PfkA	0.041 mM	-	Hellinga and Evans, 1987	no
K_M ATP of PfkA	0.2 mM	-	Zheng and Kemp, 1995	no
K_M ADP of PfkA	0.025 mM	0.2 (chosen)	Blangy and Monod, 1968	yes
K_M F6P of PfkB	0.052 mM	0.2 (chosen)	Parducci et al., 2006	yes
K_M F6P of PfkB	0.013 mM	-	Babul, 1978	no
K_M F6P of PfkB	0.009 mM	-	Baez et al., 2013	no
K_M F6P of PfkB	0.032 mM	-	Campos et al., 1984	no
K_M F6P of PfkB	0.011 mM	-	Kotlarz and Buc, 1981	no
K_M FDP of PfkB	0.14 mM	0.2 (chosen)	Babul, 1978	yes
K_M FDP of PfkB	15 mM	1.5 (chosen)	Denis' model or unknown	no

K_M ATP of PfkB	0.015 mM	0.5 (chosen)	Parducci et al., 2006	yes
K_M ATP of PfkB	0.008 mM	0.003 mM	Baez et al., 2013	no
K_M ATP of PfkB	0.020 mM	-	Campos et al., 1984	no
K_M ATP of PfkB	0.050 mM	-	Kotlarz and Buc, 1981	no
K_M ADP of PfkB	0.025 mM	0.2 (chosen)	Blangy and Monod, 1968	yes
K_D T PEP of PfkA (and PfkB)	0.75 mM	1.0 (chosen)	Blangy and Monod, 1968	yes
K_I PEP of PfkA	1.953 mM	-	Ogawa et al., 2007	no
K_I PEP of PfkA	0.30 mM	0.01 mM	Paricharttanakul et al., 2005	no
K_I ADP of PfkA	0.048 mM	0.002 mM	Paricharttanakul et al., 2005	no
K_I FDP of PfkB	0.24 mM	-	Campos et al., 1984	no
K_I ATP of PfkB	1.026 mM	0.1 mM	Baez et al., 2013	no

Glycolysis - FBP

Reaction equation

$$1\ FDP + 1\ H_2O \rightarrow 1\ F6P + 1\ Pi$$

fructose 1,6-bisphosphate + H_2O = fructose 6-phosphate + Pi

BIGG: -1 fdp_c (-1 h2o_c) +1 f6p_c +1 pi_c (gluconeogenic direction)

Enzymes and genes

EcoCyc and UniProt

FBP (fructose bisphosphatase) encoded by the *fbp* gene is part of the gluconeogenesis pathway and is required for growth on acetate, glycerol, or succinate. Fbp (FBP I or FBPA) is a homotetrameric enzyme located in the cytosol. Mg^{2+} is a cofactor for both FBP I and II. Research if Δ*fbp* mutant is viable, because 77% silencing efficacy resulted in a defect in carbon catabolite repression. Another FBP (FBP II or FBPB) is a homodimeric enzyme encoded by the *glpX* gene with lower specific activity and kinetic properties are available (EcoCyc and UniProt). The FBP of *fbp* is a class I and the FBP of *glpX* is a class II and the two enzymes share only 10% amino acid sequence similarity.

Regulation

Gene regulation: none or unknown for FBP I. cAMP binds to CRP (DNA-binding transcription factor) to enable activation and CRP activates transcription initiation of *glpX*. Glycerol binds to GlpR (DNA-binding transcription factor) to block inhibition, while GlpR inhibits transcription initiation of *glpX*, thus an increase in glycerol increases transcription of *glpX*.

Activators: Activation of FBP I by three-carbon carboxylic acids, especially by PEP (phosphoenolpyruvate) and citrate for lower concentrations of up to 5 mM which bind to the active R-state (Hines et al, 2006 and Hines and Fromm et al., 2007, respectively), likely by binding to the allosteric activator site (Hines et al., 2006). Both PEP (phosphoenolpyruvate) and citrate antagonise/block the inhibition of AMP (Babel and Guixé, 1983 and Hines and Fromm et al., 2007). (Allosteric?) activation of FBP II by PEP (Donahue et al., 2000).

Inhibitors: Allosteric inhibition of FBP I by AMP (Babul and Guixé, 1983) and G6P (glucose 6-phosphate; Hines and Kreusel et al., 2007) at distinct sites of the enzyme in a synergistic manner where the Fbp undergoes a transition into a T-like inactive state. Substrate inhibition of FBP I by FDP (fructose 1,6-bisphosphate) at concentrations above 0.05 mM (Babel and Guixé, 1983). Competitive (product) inhibition of FBP II by Pi (Donahue et al., 2000 and Brown et al., 2009) and by F1P. Inhibition of FBP II by ADP (Donahue et al., 2000) and Li^+ (Brown et al., 2009). Possible inhibition by fructose 2,6-bisphosphate (which is not present in bacteria; Marcus et al., 1984).

Reaction and enzyme constants

Parameter	Value	Uncertainty	Reference	In current model
Reversible	no	-	-	-
k_{cat} FBP I	14.6 1/s	0.8 1/s	Kelley-Loughnane et al., 2002	no
k_{cat} FBP I	24.0 1/s	3 1/s; 0.2 (chosen)	Hines and Fromm et al., 2007	yes
k_{cat} FBP I	20.0 1/s	1 1/s	Iancu et al., 2005	no
k_{cat} FBP II	2.2 1/s	-	Donahue et al., 2000 (calculated from specific activity)	no
k_{cat} FBP II	5.7 1/s	0.1 1/s; 0.2 (chosen)	Brown et al., 2009	yes
Formation energy FDP	-2194.8 kJ/mol	2.1 kJ/mol	eQuilibrator	yes
Formation energy F6P	-1302.1 kJ/mol	1.3 kJ/mol	eQuilibrator	yes
Formation energy Pi	-1072.6 kJ/mol	1.5 kJ/mol	eQuilibrator	yes
K_M FDP of FBP I	0.0154 mM	0.002 mM	Kelley-Loughnane et al., 2002	no
K_M FDP of FBP I	0.0017 mM	0.0001 mM; 0.2 (chosen)	Hines and Fromm et al., 2007	yes
K_M FDP of FBP I	0.0012 mM	0.00005 mM	Iancu et al., 2005	no
K_M F6P of FBP I	0.69 mM	1.5 (chosen)	Denis' model or unknown	no
K_M F6P of FBP I	0.02 mM	0.5 (chosen)	Picked based on previous simulation	yes
K_M Pi of FBP I	1.0 mM	1.5 (chosen)	Denis' model or unknown	no
K_M Pi of FBP I	0.02 mM	0.5 (chosen)	Picked based on previous simulation	yes
K_M FDP of FBP II	0.035 mM	-	Donahue et al., 2000	no
K_M FDP of FBP II	0.07 mM	0.002 mM; 0.2 (chosen)	Brown et al., 2009	yes
K_M F6P of FBP II	0.02 mM	0.5 (chosen)	Picked based on previous simulation of FBP I	yes
K_M Pi of FBP II	0.02 mM	0.5 (chosen)	Picked based on previous simulation of FBP I	yes
K_D R ($A_{0.5}$) PEP of FBP I	0.040 mM	-	Hines et al, 2006	no
K_D R ($A_{0.5}$) PEP of FBP I	0.027 mM	0.002 mM; 1.0 (chosen)	Hines and Fromm et al., 2007	yes
K_D R ($A_{0.5}$) CIT of FBP I	0.21 mM	0.01 mM	Hines and Fromm et al., 2007	no
K_I ($I_{0.5}$) AMP of FBP I	0.0181 mM	0.0005 mM	Hines and Fromm et al., 2007	no
K_I ($I_{0.5}$) AMP of FBP I	0.008 mM	0.002 mM	Hines and Kreusel et al., 2007	no
K_I AMP of FBP I	0.0006 mM	0.0001 mM	Iancu et al., 2005	no
K_D T ($I_{0.5}$) G6P of FBP I	0.038 mM	0.006 mM; 1.0 (chosen)	Hines and Kreusel et al., 2007	yes

Glycolysis - FBA

Reaction equation

$1\ FDP \rightarrow 1\ DHAP + 1\ G3P$

fructose 1,6-biphosphate = dihydroxyacetone phosphate + glyceraldehyde 3-phosphate

BIGG: -1 fdp_c +1 dhap_c +1 g3p_c (glycolytic direction)

Enzymes and genes

EcoCyc FbaA, EcoCyc FbaB and UniProt FbaA, UniProt FbaB

The FBA (fructose bisphosphate aldolase) reaction consists of two isozymes: FbaA (FBA class II) encoded by the *fbaA* gene and FbaB (FBA class I) encoded by the *fbaB* gene. Both enzymes are present in the cytosol, however only 5% to 10% of the total reaction activity can be attributed to FbaB in general and FbaB is only present when grown on three-carbon substrates such as lactate or glycerol, while FbaA is always present (Scamuffa and Caprioli, 1980). Thus, FbaA is likely involved in glycolysis and FbaB in gluconeogenesis. FbaA is a homodimeric enzyme, while FbaB is a homodecameric enzyme. A Δ*fbaA* mutant is viable, but has a heat-sensitive defect in rRNA transcription. Zn^{2+} is a cofactor of FbaA. Different specific activities of FBA when grown on glucose or glycerol (Szwergold et al., 1995).

Regulation

Gene regulation: Fructofuranose 1-phosphate binds to Cra (DNA-binding transcription factor) to block inhibition, while Cra inhibits transcription initiation of *fbaA* and of *fbaB*, thus an increase in fructofuranose 1-phosphate increases transcription of *fbaA* and of *fbaB*. cAMP binds to CRP (DNA-binding transcription factor) to enable activation and CRP activates transcription initiation of *fbaA*. ppGpp activates binding of RNA polymerase to the *fbaB* promotor under the condition of isoleucine starvation.

Activators: Activation of FbaA by NH_4 (ammonium) and K^+ (Blom et al., 1996). Activation of FbaB by citrate (Thomson et al., 1998; kinetic values with activation in paper) and PEP (phosphoenolpyruvate; Baldwin and Perham, 1978).

Inhibitors: Inhibition of FbaA by Ni^{2+} (nickel; Macomber et al., 2011) and product inhibition of FbaA by DHAP (competitive; dihydroxyacetone phosphate; Plater et al., 1999) and G3P (uncompetitive). Competitive inhibition of FbaA by 2-phosphoglycolate (2PGly; Qamar et al., 1996). Non-competitive inhibition of FbaB by PEP (Ogawa et al., 2007). Irreversible inhibition of FbaB by borohydride (Thomson et al., 1998).

Reaction and enzyme constants

Parameter	Value	Uncertainty	Reference	In current model
Reversible	yes	-	-	-
k_{cat} FbaA	10.5 1/s	0.07 1/s; 0.2 (chosen)	Zgiby et al., 2002 (WT)	no
k_{cat} FbaA	14.2 1/s	0.6 1/s	Hao and Berry, 2004	no
k_{cat} FbaA	8.2 1/s	0.4 1/s	Qamar et al., 1996	no
k_{cat} FbaA	10.3 or 30.3 1/s	0.3 or 0.8 1/s; 0.2 (chosen)	Berry and Marshall, 1993	yes
k_{appt}^{max} x2 FBA	55.5 x2 = 111.0 1/s	-	Calculated from experimental fluxes and protein concentrations (ΔsdhCB)	no
k_{cat} FbaB	4.14 1/s	0.2 (chosen)	Smallbone et al., 2013 (yeast)	no
k_{cat} FbaB	0.23 1/s	0.007 1/s; 0.2 (chosen)	Thomson et al., 1998	yes
Formation energy FDP	-2194.8 kJ/mol	2.1 kJ/mol	eQuilibrator	yes
Formation energy DHAP	-1097.2 kJ/mol	1.1 kJ/mol	eQuilibrator	yes
Formation energy G3P	-1091.5 kJ/mol	1.3 kJ/mol	eQuilibrator	yes
K_M FDP of FbaA	0.17 mM	0.003 mM	Zgiby et al., 2002 (WT)	no
K_M FDP of FbaA	0.133 mM	0.5 (chosen)	Babul et al., 1993	no
K_M FDP of FbaA	0.85 mM	-	Stribling and Perham, 1973	no
K_M FDP of FbaA	0.14 mM	0.02 mM	Hao and Berry, 2004	no
K_M FDP of FbaA	0.19 mM	0.03 mM	Qamar et al., 1996	no
K_M FDP of FbaA	(0.24 or) 0.29 mM	(0.03 or) 0.04 mM; 0.2 (chosen)	Berry and Marshall, 1993	yes
K_M DHAP of FbaA	0.03 mM	0.002 mM	Zgiby et al., 2002 (WT)	no
K_M DHAP of FbaA	0.088 mM	0.2 (chosen)	Babul et al., 1993	yes
K_M G3P of FbaA	0.088 mM	0.2 (chosen)	Babul et al., 1993	yes
K_M FDP of FbaB	0.451 mM	0.5 (chosen)	Smallbone et al., 2013 (yeast)	no
K_M FDP of FbaB	0.055 mM	0.003 mM	Ogawa et al., 2007	no
K_M FDP of FbaB	0.02 mM	0.002 mM; 0.2 (chosen)	Thomson et al., 1998	yes
K_M FDP of FbaB	0.02 mM	-	Stribling and Perham, 1973	no
K_M DHAP of FbaB	2.0 mM	0.5 (chosen)	Smallbone et al., 2013 (yeast)	yes
K_M G3P of FbaB	2.4 mM	0.5 (chosen)	Smallbone et al., 2013 (yeast)	yes
K_I DHAP of FbaA	0.13 mM	0.011 mM	Plater et al., 1999	no
K_I G3P of FbaA	0.6 mM	-	Babul et al., 1993	no
K_I 2PGly of FbaA	0.009 mM	0.0012 mM	Qamar et al., 1996	no
K_I PEP of FbaB	1.85 mM	-	Ogawa et al., 2007	no

Glycolysis - TPI

Reaction equation

$1\ DHAP \rightarrow 1\ G3P$

dihydroxyacetone phosphate = glyceraldehyde 3-phosphate

BIGG: -1 dhap_c +1 g3p_c

Enzymes and genes

EcoCyc and UniProt

TPI (triosephosphate isomerase) is a homodimeric enzyme encoded by the *tpiA* gene and located in the cytosol. A Δ*tpiA* mutant is viable, but was unable to grow on glucose, lactate, or other carbon sources that require the activity of both the glycolysis and gluconeogenesis pathways. Expression of *tpiA* is upregulated in a Δ*pgi* mutant and downregulated in a Δ*pykF* mutant.

Regulation

Gene regulation: Fructofuranose 1-phosphate binds to Cra (DNA-binding transcription factor) to block inhibition, while Cra inhibits transcription initiation of *tpiA*, thus an increase in fructofuranose 1-phosphate increases transcription of *tpiA*.

Activators: Activation by CrsA (carbon storage regulator; Sabnis et al., 1995).

Inhibitors: Competitive inhibition by 2-phosphoglycolate (Mainfroid et al., 1993).

Reaction and enzyme constants

Parameter	Value	Uncertainty	Reference	In current model
Reversible	yes	-	-	-
k_{cat}	600.0 1/s	0.2 (chosen)	Hermes et al., 1990 (muscle enzyme)	no
k_{cat}	8700.0 1/s	0.2 (chosen)	Mainfroid et al., 1993	yes
Formation energy DHAP	-1097.2 kJ/mol	1.1 kJ/mol	eQuilibrator	yes
Formation energy G3P	-1091.5 kJ/mol	1.3 kJ/mol	eQuilibrator	yes
K_M DHAP	0.65 mM	0.5 (chosen)	Hermes et al., 1990 (muscle enzyme)	no
K_M DHAP	2.8 mM	0.2 (chosen)	Babul et al., 1993	yes
K_M G3P	0.42 mM	0.5 (chosen)	Hermes et al., 1990 (muscle enzyme)	no
K_M G3P	1.03 mM	1.0 (chosen)	Mainfroid et al., 1993	no
K_M G3P	0.3 mM	0.2 (chosen)	Babul et al., 1993	yes
K_I 2-PG	0.006 mM	-	Mainfroid et al., 1993	no

Glycolysis - GAPD

Reaction equation

$1\ G3P + 1\ NAD + 1\ Pi \rightarrow 1\ 13DPG + 1\ NADH$

glyceraldehyde 3-phosphate + NAD + Pi = 3-phosphoglyceroyl phosphate + NADH

BIGG: -1 g3p_c -1 nad_c -1 pi_c +1 13dpg_c +1 nadh_c (glycolytic direction)

Enzymes and genes

EcoCyc and UniProt

GAPD (glyceraldehyde 3-phosphate dehydrogenase) is an homotetrameric enzyme encoded by *gapA* gene and is located in the cytosol. A Δ*gapA* mutant with 93% efficacy is viable, but exhibits a severe growth defect. Both GAPD and E4PD (erythrose 4-phosphate dehydrogenase) are able to phosphorylise G3P and E4P, however GAPD is only highly efficient for G3P phosphorylation and E4PD is only highly efficient for E4P phosphorylation.

Regulation

Gene regulation: cAMP binds to CRP (DNA-binding transcription factor) to enable activation and CRP activates transcription initiation of *gapA*. Fructofuranose 1-phosphate binds to Cra (DNA-binding transcription factor) to block inhibition, while Cra inhibits transcription initiation of *gapA*, thus an increase in fructofuranose 1-phosphate increases transcription of *gapA*.

Activators: Activation by arsenate (Zhao et al., 1995).

Inhibitors: Inhibition by iodoacetate (D'Alessio and Josse, 1971).

Reaction and enzyme constants

Parameter	Value	Uncertainty	Reference	In current model
Reversible	yes	-	-	-
k_{cat}	1056.0 1/s	0.2 (chosen)	Soukri et al., 1989	yes
k_{cat}	268.0 1/s	6 1/s	Eyschen et al., 1999	no
k_{cat}	76.0 1/s	4 1/s	Boschi-Muller et al., 1997 (*B. stearothermophilus*)	no
Formation energy G3P	-1091.5 kJ/mol	1.3 kJ/mol	eQuilibrator	yes
Formation energy 13DPG	-2212.0 kJ/mol	2.1 kJ/mol	eQuilibrator	yes
Formation energy Pi	-1072.6 kJ/mol	1.5 kJ/mol	eQuilibrator	yes
Formation energy NAD^+	-1146.0 kJ/mol	13.1 kJ/mol	eQuilibrator	yes
Formation energy NADH	-1079.8 kJ/mol	13.1 kJ/mol	eQuilibrator	yes
K_M G3P	1.5 mM	0.5 (chosen)	Soukri et al., 1989	no
K_M G3P	0.89 mM	0.17 mM; 0.5 (chosen)	Eyschen et al., 1999	yes
K_M G3P	0.90 mM	0.20 mM	Boschi-Muller et al., 1997 (*B. stearothermophilus*)	no
K_M 13DPG	0.015 mM	0.5 (chosen)	Soukri et al., 1989	yes
K_M 13DPG	0.005 mM	-	Boschi-Muller et al., 1997 (*B. stearothermophilus*)	no
K_M Pi	22.0 mM	1.0 (chosen)	Soukri et al., 1989	no
K_M Pi	0.53 mM	0.11 mM; 0.5 (chosen)	Eyschen et al., 1999	yes
K_M NAD^+	0.042 mM	0.5 (chosen)	Soukri et al., 1989	no
K_M NAD^+	0.045 mM	0.004 mM; 0.5 (chosen)	Eyschen et al., 1999	yes
K_M NAD^+	0.09 mM	0.01 mM	Boschi-Muller et al., 1997 (*B. stearothermophilus*)	no
K_M NADH	0.011 mM	0.5 (chosen)	Boschi-Muller et al., 1997 (*B. stearothermophilus*)	yes

Glycolysis - PGK

Reaction equation

$1\ 13DPG + 1\ ADP \rightarrow 1\ 3PG + 1\ ATP$

3-phosphoglyceroyl phosphate + ADP = 3-phosphoglycerate + ATP

BIGG: -1 3pg_c -1 atp_c +1 13dpg_c +1 adp_c (gluconeogenic direction)

Enzymes and genes

EcoCyc and UniProt

PGK (phosphoglycerate kinase) is an monomeric enzyme encoded by the *pgk* gene and is located in the cytosol. A Δ*pgk* mutant cannot grow on sugars or gluconeogenic substrates, but a mutant silenced with 79% efficacy did not cause severe growth inhibition. Mg^{2+} is a cofactor.

Regulation

Gene regulation: cAMP binds to CRP (DNA-binding transcription factor) to enable activation and CRP activates transcription initiation of *pgk*. Fructofuranose 1-phosphate binds to Cra (DNA-binding transcription factor) to block inhibition, while Cra inhibits transcription initiation of *pgk*, thus an increase in fructofuranose 1-phosphate increases transcription of *pgk*.

Activators: Activation by Na_2SO_4 (sodium sulfate; Fifes and Scopes., 1978).

Inhibitors: none or unknown.

Reaction and enzyme constants

Parameter	Value	Uncertainty	Reference	In current model
Reversible	yes	-	-	-
k_{cat}	349.6 1/s	0.2 (chosen)	Fifes and Scopes., 1978 (calculated from specific activity of gluconeogenic reaction)	yes
k_{cat}	1480.0 1/s	-	Collinet et al., 2000	no
k_{cat}	571.7 1/s	-	Kuntz and Krietsch, 1982 (from yeast; calculated from specific activity))	no
k_{appt}^{max} x2	107.4 x2 = 214.8 1/s	0.2 (chosen)	Calculated from experimental fluxes and protein concentrations (ΔsdhCB)	no
Formation energy 13DPG	-2212.0 kJ/mol	2.1 kJ/mol	eQuilibrator	yes
Formation energy 3PG	-1356.7 kJ/mol	1.5 kJ/mol	eQuilibrator	yes
Formation energy ATP	-2280.7 kJ/mol	2.9 kJ/mol	eQuilibrator	yes
Formation energy ADP	-1405.9 kJ/mol	2.4 kJ/mol	eQuilibrator	yes
K_M 13DPG	0.0018 mM	0.5 (chosen)	Kuntz and Krietsch, 1982 (from yeast)	yes
K_M 3PG	0.69 or 1.28 mM	0.5 (chosen)	Kuntz and Krietsch, 1982 (from yeast)	yes
K_M ATP	0.48 mM	0.5 (chosen)	Kuntz and Krietsch, 1982 (from yeast)	yes
K_M ATP	0.24 mM	-	Fifes and Scopes., 1978	no
K_M ADP	0.2 mM	0.5 (chosen)	Kuntz and Krietsch, 1982 (from yeast)	yes

Glycolysis - PGM

Reaction equation

$1\ 3PG \rightarrow 1\ 2PG$

3-phosphoglycerate = glycerate 2-phosphate

BIGG: -1 2pg_c +1 3pg_c (gluconeogenic direction)

Enzymes and genes

EcoCyc dPgm, EcoCyc iPgm and UniProt dPgm, UniProt iPgm

The PGM (phosphoglycerate mutase) reaction consists of two isozymes: dPgm encoded by *gpmA* gene and iPgm encoded by *gpmM* gene. Both enzymes are present in the cytosol and expressed at high levels, but dPgm has a 10-fold higher specific activity than iPgm (Fraser et al., 1999). dPgm is a homodimeric enzyme which needs 2,3-bisphosphoglycerate as cofactor (hence the name 2,3-bisphosphoglycerate-dependent Pgm), while iPgm is a monomeric enzyme which needs Mn^{2}+ as cofactor. A Δ*gpmA* mutant is viable, but shows a growth lag in minimal medium. A Δ*gpmM* mutant is viable, while a double mutant, Δ*gpmA*Δ*gpmM*, does not appear to be viable.

Regulation

Gene regulation: Fe^{2+} binds Fur (DNA-binding transcription factor) to enable inhibition of transcription initiation *of gpmA*. Fructofuranose 1-phosphate binds to Cra (DNA-binding transcription factor) to block inhibition, while Cra inhibits transcription initiation of *gpmM*, thus an increase in fructofuranose 1-phosphate increases transcription of *gpmM*.

Activators: none or unknown.

Inhibitors: Inhibition of dPgm by potassium and phosphate (García-Contreras et al., 2012) and by vanadate (Fraser et al., 1999; includes K_I values for both competitive and uncompetitive inhibition).

Reaction and enzyme constants

Parameter	Value	Uncertainty	Reference	In current model
Reversible	yes	-	-	-
k_{cat} dPgm	330.0 1/s	11 1/s	Fraser et al., 1999; glycolytic	no
k_{cat} dPgm	220.0 1/s	13 1/s, 0.2 (chosen)	Fraser et al., 1999; gluconeogenic	yes
k_{cat} iPgm	22.0 1/s	1 1/s	Fraser et al., 1999; glycolytic	no
k_{cat} iPgm	10.0 1/s	0.5 1/s	Fraser et al., 1999; gluconeogenic	no
Formation energy 3PG	-1356.7 kJ/mol	1.5 kJ/mol	eQuilibrator	yes
Formation energy 2PG	-1352.2 kJ/mol	1.6 kJ/mol	eQuilibrator	yes
K_M 3PG of dPgm	0.20 mM	0.027 mM; 0.5 (chosen)	Fraser et al., 1999	yes
K_M 3PG of iPgm	0.21 mM	0.039 mM	Fraser et al., 1999	no
K_M 2PG of dPgm	0.19 mM	0.035 mM; 0.5 (chosen)	Fraser et al., 1999	yes
K_M 2PG of iPgm	0.097 mM	0.014 mM	Fraser et al., 1999	no

*Only proteomics data for dPgm.

Glycolysis - ENO

Reaction equation

$1\ 2PG \rightarrow 1\ PEP + 1\ H_2O$

glycerate 2-phosphate = phosphoenolpyruvate + H_2O

BIGG: -1 2pg_c +1 pep_c (+1 h20_c) (glycolytic direction)

Enzymes and genes

EcoCyc and UniProt

ENO (enolase) is a homodimeric enzyme encoded by the *eno* gene and is located in the cytosol. A Δ*eno* mutant is viable, but glycolytic pathway intermediates accumulated when growing on glucose or glycerol, while growth on glycerate or succinate is undisturbed. Mg^{2+} is a cofactor.

Regulation

Gene regulation: L-leucine binds to Lrp (DNA-binding transcription factor) to block activation, while Lrp activates transcription initiation of *eno*, thus an increase in L-leucine decreases transcription of *eno*. Fructofuranose 1-phosphate binds to Cra (DNA-binding transcription factor) to block inhibition, while Cra inhibits transcription initiation of *eno*, thus an increase in fructofuranose 1-phosphate increases transcription of *eno*.

Activators: none or unknown.

Inhibitors: Inhibition by fluoride in the presence of phosphate (Spring and Wold, 1971).

Reaction and enzyme constants

Parameter	Value	Uncertainty	Reference	In current model
Reversible	yes	-	-	-
k_{cat}	138.0 1/s	-	Spring and Wold, 1971 (calculated from specific activity)	no
k_{cat}	199.3 1/s	10.0 1/s; 0.2 (chosen)	Kühnel and Luisi, 2001 (calculated from specific activity)	yes
k_{cat}	8.5 1/s	0.2 (chosen)	Krucinska et al., 2019	no
k_{appt}^{max} x2	52.4 x2 = 104.8 1/s	-	Calculated from experimental fluxes and protein concentrations (ΔptsHIcrr)	no
Formation energy 2PG	-1352.2 kJ/mol	1.6 kJ/mol	eQuilibrator	yes
Formation energy PEP	-1205.1 kJ/mol	1.6 kJ/mol	eQuilibrator	yes
K_M 2PG	0.1 mM	1.0 (chosen)	Spring and Wold, 1971	no
K_M 2PG	0.113 mM	0.5 (chosen)	Krucinska et al., 2019	yes
K_M PEP	0.1 mM	0.5 (chosen)	Spring and Wold, 1971	yes

PPP - G6PDH2r

Reaction equation

$$1\ G6P + 1\ NADP \rightarrow 1\ 6PGL + 1\ NADPH$$

glucose 6-phosphate + NADP = 6-phospho-glucono-1,5-lactone + NADPH

BIGG: -1 g6p_c -1 nadp_c +1 6pgl_c +1 nadph_c

Enzymes and genes

EcoCyc and UniProt

G6PDH2r (glucose 6-phosphate dehydrogenase) is a monomeric enzyme encoded by the *zwf* gene and located in the cytosol. G6PDH2r has a strong preference for $NADP^+$ over NAD^+ (Olavarría et al., 2012). A Δ*zwf* mutant grows, but the metabolic flux through CCM is altered which is why the *zwf* gene is a common metabolic engineering target. (oxidative branch)

Regulation

Gene regulation: MarA, Rob, and SoxS (DNA-binding transcription factors) activate transcription initiation of *zwf*. Fe^{2+} binds Fur (DNA-binding transcription factor) to enable inhibition of transcription initiation of *zwf* and FNR (DNA-binding transcription factors) inhibits transcription initiation of *zwf*. Fructofuranose 1-phosphate binds to Cra (DNA-binding transcription factor) to block inhibition, while Cra inhibits transcription initiation of *zwf*, thus an increase in fructofuranose 1-phosphate increases transcription of *zwf*.

Activators: none or unknown.

Inhibitors: Competitive product inhibition by NADPH to $NADP^+$ and not to G6P (Sanwal, 1970; Olavarría et al., 2012; Christodoulou et al., 2018). Allosteric inhibition by NADH (Sanwal, 1970).

Reaction and enzyme constants

Parameter	Value	Uncertainty	Reference	In current model
Reversible	no	-	-	-
k_{cat}	174.0 1/s	2 1/s; 0.2 (chosen)	Olavarría et al., 2012	yes
k_{cat}	178.0 1/s	-	Fuentealba et al., 2016	no
k_{appt}^{max} x2	107.5 x2 = 215.0 1/s	-	Calculated from experimental fluxes and protein concentrations (ΔptsHIcrr)	no
Formation energy G6P	-1304.7 kJ/mol	1.3 kJ/mol	eQuilibrator	yes
Formation energy 6PGL	-1377.7 kJ/mol	2.9 kJ/mol	eQuilibrator	yes
Formation energy $NADP^+$	-2033.6 kJ/mol	13.4 kJ/mol	eQuilibrator	yes
Formation energy NADPH	-1967.4 kJ/mol	13.4 kJ/mol	eQuilibrator	yes
K_M G6P	0.07 mM	-	Sanwal, 1970	no
K_M G6P	0.145 mM	-	Banerjee and Fraenkel, 1972	no
K_M G6P	0.2 mM	-	Westwood and Doelle, 1974	no
K_M G6P	0.174 mM	0.011 mM; 0.5 (chosen)	Olavarría et al., 2012	yes
K_M 6PGL	0.122 mM	1.0 (chosen)	Estimated from Banerjee and Fraenkel, 1972	yes
K_M $NADP^+$	0.015 mM	-	Sanwal, 1970	no
K_M $NADP^+$	0.015 mM	-	Banerjee and Fraenkel, 1972	no
K_M $NADP^+$	0.02 mM	-	Westwood and Doelle, 1974	no
K_M $NADP^+$	0.0075 mM	0.0008 mM; 0.5 (chosen)	Olavarría et al., 2012	yes
K_M $NADP^+$	0.008 mM	-	Fuentealba et al., 2016	no
K_M NADPH	0.0168 mM	1.0 (chosen)	Estimated from Banerjee and Fraenkel, 1972	yes
K_I NADPH or K_D T NADPH	0.035 mM	0.5 (chosen)	Christodoulou et al., 2018	no
K_I NADPH	0.014 mM	0.002 mM	Olavarría et al., 2012, but estimated by Christodoulou et al., 2018	no
K_D T NADH	0.05 mM	1.0 (chosen)	Estimated from Lineweaver-Burk plot in Sanwal, 1970	no
K_D T 6PGC	0.05 mM	1.5 (chosen)	Realistic estimate of tense dissociation constant with large scale	yes

PPP - PGL

Reaction equation

$1\ 6PGL + 1\ H_2O \rightarrow 1\ 6PGC$

6-phospho-glucono-1,5-lactone + H_2O = 6-phosphogluconate

BIGG: -1 6pgl_c (-1 h2o_c) +1 6pgc_c

Enzymes and genes

EcoCyc and UniProt

PGL (6-phosphogluconolactonase) is a monomeric enzyme encoded by the *pgl* gene and located in the cytosol. A Δ*pgl* mutant is viable, but has a decreased growth rate (Kupor and Fraenkel, 1969; Kupor and Fraenkel, 1972). Non-enzymatic hydrolysis is possible, but much slower ($1.8 \cdot 10^{-4}$ 1/s; Bauer et al., 1983). (oxidative branch)

Regulation

Gene regulation: L-leucine binds to Lrp (DNA-binding transcription factor) to block inhibition of transcription initiation of *pgl*.

Activators: none or unknown.

Inhibitors: none or unknown. (The *Zymomonas mobilis* enzyme is inhibited by G6P; Scopes, 1985)

Reaction and enzyme constants

Parameter	Value	Uncertainty	Reference	In current model
Reversible	no	-	-	-
k_{cat}	2070 1/s	-	Scopes, 1985 (*Zymomonas mobilis* enzyme)	no
k_{cat}	14.3 1/s	-	Bauer et al., 1983 (human enzyme)	no
k_{cat}	472.0 1/s	0.2 (chosen)	Zimenkov et al., 2005 (calculated from specific activity)	yes
k_{appt}^{max} x2	248.0 x2 = 496.0 1/s	0.2 (chosen)	Calculated from experimental fluxes and protein concentrations (ΔsdhCB)	no
Formation energy 6PGL	-1377.7 kJ/mol	2.9 kJ/mol	eQuilibrator	yes
Formation energy 6PGC	-1554.0 kJ/mol	2.4 kJ/mol	eQuilibrator	yes
K_M 6PGL	0.023 mM	0.002 mM; 1.0 (chosen)	Scopes, 1985 (*Zymomonas mobilis* enzyme)	yes
K_M 6PGL	0.83 mM	-	Bauer et al., 1983 (human enzyme)	no
K_M 6PGC	10 mM	1.0 (chosen)	Scopes, 1985 (*Zymomonas mobilis* enzyme)	yes

PPP - GND

Reaction equation

$$1\ 6PGC + 1\ NADP \rightarrow 1\ RU5P + 1\ NADPH + 1\ CO_2$$

6-phospho-gluconate (gluconate 6-phosphate) + NADP = ribulose 5-phosphate + NADPH + CO_2

BIGG: -1 6pgc_c -1 nadp_c +1 ru5p_D_c +1 nadph_c +1 co2_c

Enzymes and genes

EcoCyc and UniProt

GND (phosphogluconate dehydrogenase) is a homodimeric enzyme encoded by the *gnd* gene and located in the cytosol. A Δ*gnd* mutant is viable, but has a decreased growth rate (Jiao et al., 2003; Zhao et al., 2004). GND cannot use NAD^+ in place of $NADP^+$ for the reaction. Reported conformational changes upon binding of $NADP^+$ (Chen et al., 2010). (oxidative branch)

Regulation

Gene regulation: GadE (DNA-binding transcription factors) activates transcription initiation of *gnd*. Fe^{2+} binds Fur (DNA-binding transcription factor) to enable inhibition of transcription initiation of *gnd* and FNR (DNA-binding transcription factors) inhibits transcription initiation of *gnd*. Expression is strongly coupled to the growth rate.

Activators: none or unknown.

Inhibitors: Competitive inhibition by ATP to 6PGC and NADP (De Silva and Fraenkel, 1979) and competitive product inhibition by NADPH to NADP (Westwood and Doelle, 1974). Product inhibition by RU5P (De Silva and Fraenkel, 1979) and (allosteric) inhibition by FDP (Wolf and Shea, 1979). Possible inhibition by PEP (Peskov et al., 2012)

Reaction and enzyme constants

Parameter	Value	Uncertainty	Reference	In current model
Reversible	no	-	-	-
k_{cat}	21.1 1/s	0.2 (chosen)	Chen et al., 2010	no
k_{cat}	27.5 1/s	-	Veronese et al., 1976 (based on specific activity)	no
k_{appt}^{max}	63.7 1/s	0.2 (chosen)	Calculated from experimental fluxes and protein concentrations (Δpgi)	yes
Formation energy 6PGC	-1554.0 kJ/mol	2.4 kJ/mol	eQuilibrator	yes
Formation energy RU5P	-1223.9 kJ/mol	1.9 kJ/mol	eQuilibrator	yes
Formation energy $NADP^+$	-2033.6 kJ/mol	13.4 kJ/mol	eQuilibrator	yes
Formation energy NADPH	-1967.4 kJ/mol	13.4 kJ/mol	eQuilibrator	yes
Formation energy CO_2	-403.1 kJ/mol	5.7 kJ/mol	eQuilibrator	yes
K_M 6PGC	0.026 mM	-	Wolf and Shea, 1979	no
K_M 6PGC	0.05 mM	-	Veronese et al., 1976	no
K_M 6PGC	0.093 mM	0.001 mM; 0.5 (chosen)	Chen et al., 2010	yes
K_M RU5P	0.044 mM	0.5 (chosen)	Estimated from De Silva and Fraenkel, 1979	yes
K_M $NADP^+$	0.011 mM	-	Wolf and Shea, 1979	no
K_M $NADP^+$	0.033 mM	-	Veronese et al., 1976	no
K_M $NADP^+$	0.049 mM	0.007 mM; 0.5 (chosen)	Chen et al., 2010	yes
K_M NADPH	0.0034 mM	0.5 (chosen)	Estimated from De Silva and Fraenkel, 1979	yes
K_M CO_2	0.1 mM	2.0 (chosen)	unknown	yes
K_I FDP	0.025 mM	-	De Silva and Fraenkel, 1979	no

PPP - RPI

Reaction equation

$1\ RU5P \rightarrow 1\ R5P$

ribulose 5-phosphate = ribose 5-phosphate

BIGG: -1 r5p_c +1 ru5p_D_c

Enzymes and genes

EcoCyc RpiA, EcoCyc RpiB and UniProt RpiA, UniProt RpiB

The RPI (ribose-5-phosphate isomerase) reaction consists of two isozymes: RpiA encoded by the *rpiA* gene and RpiB encoded by the *rpiB* gene. Both isozymes are present in the cytosol, however more than 99% of the total reaction activity in the wild type can be attributed to RpiA (Skinner and Cooper, 1971). RpiA is constitutive while RpiB is only present when induced by allose (RpiB is also involved in allose catabolism), but RpiB can substitute RpiA's function (Skinner and Cooper, 1974; Sørensen and Hove-Jensen, 1996). Both enzymes are homodimeric. A Δ*rpiA* mutant requires ribose for growth, while a Δ*rpiB* mutant cannot catabolise allose. (non-oxidative branch)

Regulation

Gene regulation: L-leucine binds to Lrp (DNA-binding transcription factor) to block inhibition, while Lrp inhibits transcription initiation of *rpiA*, thus an increase in L-leucine increases transcription of *rpiA*. L-leucine binds to Lrp (DNA-binding transcription factor) to block activation, while Lrp activates transcription initiation of *rpiB*, thus an increase in L-leucine decreases transcription of *rpiB*. Glutarate binds to GlaR (DNA-binding transcription factor) to block activation, while GlaR activates transcription initiation of *rpiB*, thus an increase in glutarate decreases transcription of *rpiB*. Allose binds to AlsR (DNA-binding transcription factor) to block inhibition, while AlsR inhibits transcription initiation of *rpiB*, thus an increase in allose increases transcription of *rpiB*.

Activators: none or unknown.

Inhibitors: Competitive inhibition of RpiA by arabinose 5-phosphate (A5P), E4P (erythrose 4-phosphate), erythronic acid (EA), and 4-phosphoerythronic acid (PEA) (Zhang et al., 2003). Inhibition of RpiA by AMP (Essenberg and Cooper, 1975). Inhibition of RpiB by iodoacetate (Essenberg and Cooper, 1975) and non-competitive inhibition by G6P (glucose 6-phosphate; David and Wiesmeyer, 1970, not found in Essenberg and Cooper, 1975).

Reaction and enzyme constants

Parameter	Value	Uncertainty	Reference	In current model
Reversible	yes	-	-	-
k_{cat} RpiA	2100.0 1/s	300.0 1/s	Zhang et al., 2003	yes
k_{cat} RpiB	52.0 1/s	2 1/s	Roos et al., 2008	no
k_{appt}^{max} x2	114.6 x2 = 229.2 1/s	-	Calculated from experimental fluxes and protein concentrations (ΔptsHIcrr)	no
Formation energy RU5P	-1223.9 kJ/mol	1.9 kJ/mol	eQuilibrator	yes
Formation energy R5P	-1226.0 kJ/mol	2.0 kJ/mol	eQuilibrator	yes
K_M RU5P of RpiA	0.0035 mM	0.5 (chosen)	Estimated from Zhang et al., 2003	yes
K_M R5P of RpiA	6.2 mM	-	Skinner and Cooper, 1971	no
K_M R5P of RpiA	1.85 - 2.59 mM	-	David and Wiesmeyer, 1970	no
K_M R5P of RpiA	4.4 mM	0.5 mM	Essenberg and Cooper, 1975	no
K_M R5P of RpiA	3.1 mM	0.2 mM; 0.5 (chosen)	Zhang et al., 2003	yes
K_M RU5P of RpiB	0.0035 mM	-	Estimated from Zhang et al., 2003	no
K_M R5P of RpiB	0.95 mM	-	Skinner and Cooper, 1971	no
K_M R5P of RpiB	0.13 - 0.25 mM	-	David and Wiesmeyer, 1970	no
K_M R5P of RpiB	0.83 mM	0.13 mM	Essenberg and Cooper, 1975	no
K_M R5P of RpiB	1.1 mM	0.2 mM	Roos et al., 2008	no
K_I A5P of RpiA	2.1 mM	0.9 mM	Zhang et al., 2003	no
K_I E4P of RpiA	0.67 mM	-	Zhang et al., 2003	no
K_I EA of RpiA	0.32 mM	-	Zhang et al., 2003	no
K_I PEA of RpiA	0.004 mM	-	Zhang et al., 2003	no
K_I G6P of RpiB	0.060 - 0.085 mM	-	David and Wiesmeyer, 1970	no

PPP - RPE

Reaction equation

$1\ RU5P \rightarrow 1\ XU5P$

ribulose 5-phosphate = xylulose 5-phosphate

BIGG: -1 ru5p_D_c +1 xu5p_D_c

Enzymes and genes

EcoCyc and UniProt

RPE (ribulose 5-phosphate 3-epimerase) is a monomeric enzyme encoded by the *rpe* gene and located in the cytosol. Fe^{2+} is a cofactor and can be replaced by Mn^{2+}, Co^{2+}, and Zn^{2+}. When Fe^{2+} is used as cofactor, the RPE enzyme is vulnerable to damage by H_2O_2 (hydrogen peroxide; Sobota and Imlay, 2011). A Δ*rpe* mutant only grows on minimal medium, when ribose and xylose are provided and has a strongly impaired growth rate on glucogenic substrates (Lyngstadaas et al., 1998). (non-oxidative branch)

Regulation

Gene regulation: none or unknown.

Activators: none or unknown.

Inhibitors: none or unknown.

Reaction and enzyme constants

Parameter	Value	Uncertainty	Reference	In current model
Reversible	yes	-	-	-
k_{cat}	67000 1/s	7700 1/s	Sobota and Imlay, 2011	no
k_{cat}	3800.0 1/s	160 1/s	Chan et al., 2008: with Co^{2+}	yes
k_{appt}^{max} x2	289.1 x2 = 578.2 1/s	-	Calculated from experimental fluxes and protein concentrations (Δpgi)	no
Formation energy RU5P	-1223.9 kJ/mol	1.9 kJ/mol	eQuilibrator	yes
Formation energy XU5P	-1227.3 kJ/mol	2.4 kJ/mol	eQuilibrator	yes
K_M Ru5P	1.6 mM	0.2 mM	Sobota and Imlay, 2011	no
K_M Ru5P	1.2 mM	0.1 mM	Chan et al., 2008: with Co^{2+}	yes
K_M Ru5P	1 mM	-	Hurwitz and Horecker, 1956; *Lactobacillus pentosus*	no
K_M Ru5P	0.872 mM	-	Estimated from Hurwitz and Horecker, 1956	no
K_M XU5P	0.5 mM	0.5 (chosen)	Hurwitz and Horecker, 1956; *Lactobacillus pentosus*	yes
K_M XU5P	0.893 mM	-	Estimated from Hurwitz and Horecker, 1956	no

PPP - TKT1

Reaction equation

$1\ R5P + 1\ XU5P \rightarrow 1\ G3P + 1\ S7P$

ribose 5-phosphate + xylulose 5-phosphate = glyceraldehyde 3-phosphate + sedoheptulose 7-phosphate

BIGG: -1 r5p_c -1 xu5p_D_c +1 g3p_c +1 s7p_c

Enzymes and genes

EcoCyc TktA, EcoCyc TktB and UniProt TktA, UniProt TktB

The TKT1 (transketolase 1) reaction consists of two isozymes: TktA (TKT I) encoded by the *tktA* gene and TktB (TKT II) encoded by the *tktB* gene. Both isozymes are present in the cytosol, however TktA is responsible for the major TKT activity (Iida et al., 1993). Overexpression of *tktB* and thus overproduction of TktB suppresses the *tktA* phenotype. TktA is a homodimeric enzyme, while TktB is a monomeric enzyme. TKT mutants cannot grow on pentoses and a Δ*tktA*Δ*tktB* double mutant requires pyridoxine, aromatic amino acids, and vitamins for growth. The TKT reaction is a common metabolic engineering target. Expression of tktA and tktB is complementary, resulting in approximately constant levels of transketolase expression throughout growth. TktA and TktB have high sequence similarity. Thiamine diphosphate (ThDP) and Mn^{2+} (or Mg^{2+} or Ca^{2+} or Co^{2+}) are cofactors of TktA; one per subunit. TktA can use other substrates than in TKT1 and TKT2 (review: Schenk et al., 1998). (non-oxidative branch)

Regulation

Gene regulation: none or unknown for TktA. Phosphate binds to PhoB (DNA-binding transcription factor) to enable activation of transcription initiation of *tktB*. DksA and ppGpp (under conditions of isoleucine starvation) activate binding of RNA polymerase to the promotor.

Activators: none or unknown.

Inhibitors: Competitive inhibition of TktA by arabinose 5-phosphate (A5P; Sprenger et al., 1995).

Reaction and enzyme constants

Parameter	Value	Uncertainty	Reference	In current model
Reversible	yes	-	-	-
k_{cat} TktA	61.3 1/s	0.2 (chosen)	Sprenger et al., 1995 (calculated from specific activity)	yes
k_{cat} TktA	84.6 1/s	-	Gyamerah and Willetts, 1997 (other substrates)	no
k_{cat} TktB	61.3 1/s	-	Sprenger et al., 1995 (TktB is similar to TktA; calculated from specific activity)	no
k_{appt}^{max} x2	21.8 x2 = 43.6 1/s	-	Calculated from experimental fluxes and protein concentrations (Δpgi)	no
Formation energy R5P	-1226.0 kJ/mol	2.0 kJ/mol	eQuilibrator	yes
Formation energy XU5P	-1227.3 kJ/mol	2.4 kJ/mol	eQuilibrator	yes
Formation energy G3P	-1091.5 kJ/mol	1.3 kJ/mol	eQuilibrator	yes
Formation energy S7P	-1365.6 kJ/mol	3.7 kJ/mol	eQuilibrator	yes
K_M R5P of TktA	1.4 mM	0.5 (chosen)	Sprenger et al., 1995	yes
K_M XU5P of TktA	0.16 mM	0.5 (chosen)	Sprenger et al., 1995	yes
K_M G3P of TktA	2.1 mM	0.5 (chosen)	Sprenger et al., 1995	yes
K_M S7P of TktA	4.0 mM	0.5 (chosen)	Sprenger et al., 1995	yes
K_M R5P of TktB	1.4 mM	-	Sprenger et al., 1995 (TktB is similar to TktA)	no
K_M XU5P of TktB	0.16 mM	-	Sprenger et al., 1995 (TktB is similar to TktA)	no
K_M G3P of TktB	2.1 mM	-	Sprenger et al., 1995 (TktB is similar to TktA)	no
K_M S7P of TktB	4.0 mM	-	Sprenger et al., 1995 (TktB is similar to TktA)	no
K_I A5P of TktA	6.0 mM	-	Sprenger et al., 1995	no

PPP - TKT2

Reaction equation

$1\ E4P + 1\ XU5P \rightarrow 1\ F6P + 1\ G3P$

erythrose 4-phosphate + xylulose 5-phosphate = fructose 6-phosphate + glyceraldehyde 3-phosphate

BIGG: -1 e4p_c -1 xu5p_D_c +1 f6p_c +1 g3p_c

Enzymes and genes

EcoCyc TktA, EcoCyc TktB and UniProt TktA, UniProt TktB

The TKT1 (transketolase 2) reaction consists of two isozymes: TktA (TKT I) encoded by the *tktA* gene and TktB (TKT II) encoded by the *tktB* gene. Both isozymes are present in the cytosol, however TktA is responsible for the major TKT activity (Iida et al., 1993). Overexpression of *tktB* and thus overproduction of TktB suppresses the *tktA* phenotype. TktA is a homodimeric enzyme, while TktB is a monomeric enzyme. TKT mutants cannot grow on pentoses and a Δ*tktA*Δ*tktB* double mutant requires pyridoxine, aromatic amino acids, and vitamins for growth. The TKT reaction is a common metabolic engineering target. Expression of tktA and tktB is complementary, resulting in approximately constant levels of transketolase expression throughout growth. TktA and TktB have high sequence similarity. Thiamine diphosphate (ThDP) and Mn^{2+} (or Mg^{2+} or Ca^{2+} or Co^{2+}) are cofactors of TktA; one per subunit. TktA can use other substrates than in TKT1 and TKT2 (review: Schenk et al., 1998). (non-oxidative branch)

Regulation

Gene regulation: none or unknown for TktA. Phosphate binds to PhoB (DNA-binding transcription factor) to enable activation of transcription initiation of *tktB*. DksA and ppGpp (under conditions of isoleucine starvation) activate binding of RNA polymerase to the promotor.

Activators: none or unknown.

Inhibitors: Irreversible inhibition/inactivation of TktA by superoxide (Benov and Fridovich, 1999).

Reaction and enzyme constants

Parameter	Value	Uncertainty	Reference	In current model
Reversible	yes	-	-	-
k_{cat} TktA	133.8 1/s	0.2 (chosen)	Sprenger et al., 1995 (calculated from specific activity)	yes
k_{cat} TktA	84.6 1/s	-	Gyamerah and Willetts, 1997 (other substrates)	no
k_{cat} TktB	133.8 1/s	-	Sprenger et al., 1995 (TktB is similar to TktA; calculated from specific activity)	no
k_{appt}^{max} x2	18.8 x2 = 37.6 1/s	-	Calculated from experimental fluxes and protein concentrations (Δpgi)	no
Formation energy E4P	-1156.0 kJ/mol	3.4 kJ/mol	eQuilibrator	yes
Formation energy XU5P	-1227.3 kJ/mol	2.4 kJ/mol	eQuilibrator	yes
Formation energy F6P	-1302.1 kJ/mol	1.3 kJ/mol	eQuilibrator	yes
Formation energy G3P	-1091.5 kJ/mol	1.3 kJ/mol	eQuilibrator	yes
K_M E4P of TktA	0.09 mM	0.5 (chosen)	Sprenger et al., 1995	yes
K_M XU5P of TktA	0.16 mM	0.5 (chosen)	Sprenger et al., 1995	yes
K_M F6P of TktA	1.1 mM	0.5 (chosen)	Sprenger et al., 1995	yes
K_M G3P of TktA	2.1 mM	0.5 (chosen)	Sprenger et al., 1995	yes
K_M E4P of TktB	0.09 mM	-	Sprenger et al., 1995 (TktB is similar to TktA)	no
K_M XU5P of TktB	0.16 mM	-	Sprenger et al., 1995 (TktB is similar to TktA)	no
K_M F6P of TktB	1.1 mM	-	Sprenger et al., 1995 (TktB is similar to TktA)	no
K_M G3P of TktB	2.1 mM	-	Sprenger et al., 1995 (TktB is similar to TktA)	no

PPP - TALA

Reaction equation

$$1\ G3P + 1\ S7P \rightarrow 1\ E4P + 1\ F6P$$

glyceraldehyde 3-phosphate + sedoheptulose 7-phosphate = erythrose 4-phosphate + fructose 6-phosphate

BIGG: -1 g3p_c -1 s7p_c +1 e4p_c +1 f6p_c

Enzymes and genes

EcoCyc TalaA, EcoCyc TalaB and UniProt TalaA, UniProt TalaB

The TALA (transaldolase) reaction consists of two isozymes: TalaA encoded by the *talA* gene and TalaB encoded by the *talB* gene. Both isozymes are located in the cytosol, however only TalaB has been biochemically characterised and is likely the dominant isozyme. Both a Δ*talA* or Δ*talB* do not cause a growth defect. TalaB is a homodimeric enzyme, while TalaA is likely a monomeric enzyme. *talA* expression increases in early stationary phase just as expression of *talB* decreases. (non-oxidative branch)

Regulation

Gene regulation: Phosphate binds to PhoB (DNA-binding transcription factor) to enable activation of transcription initiation of *talA*. DksA and ppGpp (under conditions of isoleucine starvation) activate binding of RNA polymerase to the promotor. None or unknown for *talB*.

Activators: none or unknown.

Inhibitors: Competitive inhibition of TalaA by FDP to F6P and uncompetitive inhibition by FDP to E4P (Ogawa et al., 2016). Competitive inhibition of TalaB by arabinose 5-phosphate (A5P), glyceraldehyde, and phosphate (Sprenger et al., 1995).

Reaction and enzyme constants

Parameter	Value	Uncertainty	Reference	In current model
Reversible	yes	-	-	-
k_{cat} TalaA	38.0 1/s	-	Ogawa et al., 2016 (calculated from specific activity)	no
k_{cat} TalaB	46.7 1/s	0.2 (chosen)	Sprenger et al., 1995 (calculated from specific activity)	yes
k_{cat} TalaB	72.0 1/s	-	Schörken et al., 1998	no
k_{cat} TalaB	79.4 1/s	-	Schörken et al., 2001	no
k_{cat} TalaB	53.0 1/s	6 1/s	Schneider et al., 2008	no
k_{cat} TalaB	46.7 1/s	-	Ogawa et al., 2016 (calculated from specific activity)	no
k_{appt}^{max} x2	25.6 x2 = 51.2 1/s	-	Calculated from experimental fluxes and protein concentrations (WT)	no
Formation energy G3P	-1091.5 kJ/mol	1.3 kJ/mol	eQuilibrator	yes
Formation energy S7P	-1365.6 kJ/mol	3.7 kJ/mol	eQuilibrator	yes
Formation energy E4P	-1156.0 kJ/mol	3.4 kJ/mol	eQuilibrator	yes
Formation energy F6P	-1302.1 kJ/mol	1.3 kJ/mol	eQuilibrator	yes
K_M G3P of TalaA	0.038 mM	-	Sprenger et al., 1995 (TalaA is likely similar to TalaB)	no
K_M S7P of TalaA	0.285 mM	-	Sprenger et al., 1995 (TalaA is likely similar to TalaB)	no
K_M E4P of TalaA	0.14 mM	0.04 mM	Ogawa et al., 2016	no
K_M F6P of TalaA	1.5 mM	0.22 mM	Ogawa et al., 2016	no
K_M G3P of TalaB	0.038 mM	0.5 (chosen)	Sprenger et al., 1995	yes
K_M S7P of TalaB	0.285 mM	0.5 (chosen)	Sprenger et al., 1995	yes
K_M E4P of TalaB	0.09 mM	0.5 (chosen)	Sprenger et al., 1995	yes
K_M E4P of TalaB	0.295 mM	-	Schörken et al., 1998	no
K_M E4P of TalaB	0.1 mM	-	Schörken et al., 2001	no
K_M E4P of TalaB	0.084 mM	0.01 mM	Ogawa et al., 2016	no
K_M F6P of TalaB	1.2 mM	0.5 (chosen)	Sprenger et al., 1995	yes
K_M F6P of TalaB	0.94 mM	-	Schörken et al., 1998	no
K_M F6P of TalaB	1.2 mM	-	Schörken et al., 2001	no
K_M F6P of TalaB	3.0 mM	0.2 mM	Schneider et al., 2008	no
K_M F6P of TalaB	0.9 mM	0.12 mM	Ogawa et al., 2016	no
K_I FDP of TalaA	3.2 mM	-	Ogawa et al., 2016	no
K_I FDP of TalaB	2.85 mM	-	Ogawa et al., 2016	no
K_I A5P of TalaB	0.05 mM	-	Sprenger et al., 1995	no

PPP - EDD

Reaction equation

$$1\ 6PGC \rightarrow 1\ 2DDG6P + 1\ H_2O$$

6-phosphogluconate = 2-dehydro-3-deoxygluconate 6-phosphate + H_2O

BIGG: -1 6pgc_c +1 2ddg6p_c (+1 h2o_c)

Enzymes and genes

EcoCyc and UniProt

EDD (6-phosphogluconate dehydratase) is a monomeric enzyme encoded by the *edd* gene and is located in the cytosol. EDD is induced by gluconate and enables growth on gluconate. EDD requires and iron-sulfur cluster (4Fe-4S specifically) as a cofactor and is thus sensitive to superoxide and hydrogen peroxide. Reduced expression levels of the GroEL-GroES molecular chaperonin complex during steady-state growth increases the synthesis of EDD and EDA, suggesting a regulatory function. A Δ*edd* mutant grows more slowly on gluconate. Mutations in *iscS* involved in iron-sulfur cluster biosynthesis result in decreased activity of EDD and a Δ*sufABCDSE* deletion mutant shows reduced EDD activity during iron starvation. The *edd* gene is a common metabolic engineering target. The enzyme EDD has not been biochemically well characterised. (Entner-Doudoroff shunt)

Regulation

Gene regulation: Fructofuranose 1-phosphate binds to Cra (DNA-binding transcription factor) to block inhibition, while Cra inhibits transcription initiation of *edd*, thus an increase in fructofuranose 1-phosphate increases transcription of *edd*. Gluconate binds to GntR (DNA-binding transcription factor) to block inhibition, while GntR inhibits transcription initiation of *edd*, thus an increase in gluconate increases transcription of *edd*. KdgR (DNA-binding transcription factor) inhibits transcription initiation of *edd*.

Activators: none or unknown.

Inhibitors: Inhibition by superoxide (O_2^-) and hydrogen peroxide (H_2O_2), and stabilised by fluoride (Gardner and Fridovich, 1991).

Reaction and enzyme constants

Parameter	Value	Uncertainty	Reference	In current model
Reversible	no	-	-	-
k_{cat}	0.4 1/s	-	Wang and Dykhuizen, 2001 (calculated from specific activity)	no
k_{appt}^{max} x2	212.6 x2 = 425.2 1/s	-	Calculated from experimental fluxes and protein concentrations (Δtpi)	yes
Formation energy 6PGC	-1554.0 kJ/mol	2.4 kJ/mol	eQuilibrator	yes
Formation energy 2DDG6P	-1445.9 kJ/mol	4.6 kJ/mol	eQuilibrator	yes
K_M 6PGC	1.356 mM	0.1 mM	Wang and Dykhuizen, 2001	no
K_M 6PGC	0.6 mM	0.5 (chosen)	Kovachevich and Wood, 1955; *Pseudomonas putida*	yes
K_M 2DDG6P	10 mM	1.5 (chosen)	Picked because of irreversible reaction	yes

PPP - EDA

Reaction equation

$1\ 2DDG6P \rightarrow 1\ G3P + 1\ PYR$

2-dehydro-3-deoxy-gluconate 6-phosphate = glyceraldehyde 3-phosphate + pyruvate

BIGG: -1 2ddg6p_c +1 g3p_c +1 pyr_c

Enzymes and genes

EcoCyc and UniProt

EDA (2-dehydro-3-deoxy-phosphogluconate or KDPG aldolase) is a homotrimeric enzyme encoded by the *eda* gene and is located in the cytosol. EDA has two additional functionalities, however at lower activity levels. EDA is induced by gluconate and enables growth on gluconate, but is constitutively expressed. A Δ*eda* mutant cannot grow on gluconate. The *eda* gene is a common metabolic engineering target. KDPG is a toxic compound and stops growth. (Entner-Doudoroff shunt)

Regulation

Gene regulation: Fructofuranose 1-phosphate binds to Cra (DNA-binding transcription factor) to block inhibition, while Cra inhibits transcription initiation of *eda*, thus an increase in fructofuranose 1-phosphate increases transcription of *eda*. Gluconate binds to GntR (DNA-binding transcription factor) to block inhibition, while GntR inhibits transcription initiation of *eda*, thus an increase in gluconate increases transcription of *eda*. KdgR (DNA-binding transcription factor) inhibits transcription initiation of *eda*. Phosphate binds to PhoB (DNA-binding transcription factor) to enable inhibition of transcription initiation of *eda*.

Activators: none or unknown.

Inhibitors: Competitive inhibition by 6PGC (6-phosphogluconate) and product inhibition by G3P (Pouysségur and Stoeber, 1971).

Reaction and enzyme constants

Parameter	Value	Uncertainty	Reference	In current model
Reversible	no	-	-	-
k_{cat}	283.8 1/s	3.4 1/s	Fong et al., 2000	no
k_{cat}	80.0 1/s	2 1/s	Walters et al., 2008	no
k_{cat}	83.0 1/s	2 1/s; 0.2 (chosen)	Cheriyan et al., 2007	yes
k_{appt}^{max} x2	37.4 x2 = 74.8 1/s	-	Calculated from experimental fluxes and protein concentrations (Δtpi)	no
Formation energy 2DDG6P	-1445.9 kJ/mol	4.6 kJ/mol	eQuilibrator	yes
Formation energy G3P	-1091.5 kJ/mol	1.3 kJ/mol	eQuilibrator	yes
Formation energy PYR	-355.2 kJ/mol	1.5 kJ/mol	eQuilibrator	yes
K_M 2DDG6P	0.2 mM	-	Pouysségur and Stoeber, 1971	no
K_M 2DDG6P	0.35 mM	0.01 mM	Fong et al., 2000	no
K_M 2DDG6P	0.1 mM	0.02 mM	Walters et al., 2008	no
K_M 2DDG6P	0.1 mM	0.01 mM; 0.5 (chosen)	Cheriyan et al., 2007	yes
K_M G3P	10 mM	1.5 (chosen)	Wymer et al., 2001 (similar to PYR, both products)	yes
K_M PYR	10 mM	0.5 (chosen)	Wymer et al., 2001	yes
K_I 6PGC	0.8 mM	-	Pouysségur and Stoeber, 1971	no
K_I G3P	0.4 mM	-	Pouysségur and Stoeber, 1971	no

PPP - PFK_3

Reaction equation

$1\ S7P + 1\ ATP \rightarrow 1\ S17BP + 1\ ADP$

sedoheptulose 7-phosphate + ATP = sedoheptulose 1,7-bisphosphate + ADP

BIGG: -1 s7p_c -1 atp_c +1 s17bp_c +1 adp_c

Enzymes and genes

EcoCyc and UniProt same as PfkA

PFK_3 (phosphofructokinase s7p) is a promiscuous activity of PfkA. This activity was discovered when the TALA reaction was blocked by deletion of talA and talB, and by feeding either xylose or gluconate as substrate (Nakahigashi et al., 2009). The activity only occurs when S7P accumulates in the cell (e.g. blocking of the TALA reaction) and would thus not likely occur in the wild type and is better to leave out of the model (Riemer et al., 2013). This promiscuous activity is slower than the native activity, so lower affinity for S7P as substrate compared to F6P. PfkA is a homotetrameric enzyme. A Δ*pfkA* strain is viable and found to increase the NADPH production via the pentose phosphate pathway. Mg^{2+} is a cofactor of both PfkA. (sedoheptulose biphosphate bypass)

Regulation

Gene regulation: F6P (fructose 6-phosphate) binds to Cra (DNA-binding transcription factor) to block inhibition, while Cra inhibits transcription initiation of *pfkA*, thus an increase in F6P increases transcription of *pfkA*.

Activators: Allosteric activation of PfkA by ADP (also GDP; Blangy and Monod, 1968) and by F6P (Johnson and Reinhart, 1992), and (product) activation of PfkA by FDP (fructose 1,6-bisphosphate) (Blangy and Monod, 1968).

Inhibitors: Allosteric inhibition of PfkA by PEP (phosphoenolpyruvate) and by ATP (Blangy and Monod, 1968; Berger and Evans, 1991).

Reaction and enzyme constants

Parameter	Value	Uncertainty	Reference	In current model
Reversible	no	-	-	-
k_{appt}^{max} x2	67.6 x2 = 135.2 1/s	0.2 (chosen)	Calculated from experimental fluxes and protein concentrations (WT)	yes
Formation energy S7P	-1365.6 kJ/mol	3.7 kJ/mol	eQuilibrator	yes
Formation energy S17BP	-2257.1 kJ/mol	4.0 kJ/mol	eQuilibrator	yes
Formation energy ATP	-2280.7 kJ/mol	2.9 kJ/mol	eQuilibrator	yes
Formation energy ADP	-1405.9 kJ/mol	2.4 kJ/mol	eQuilibrator	yes
K_M S7P	0.16 mM	-	Karadsheh et al., 1973; rabbit liver	yes
K_M S7P	0.6 mM	-	Karadsheh et al., 1973; rabbit muscle	no
K_M S17BP	10 mM	1.5 (chosen)	Picked because of irreversible reaction	yes
K_M ATP of PfkA	0.06 mM	0.5 (chosen)	Blangy and Monod, 1968	yes
K_M ATP of PfkA	0.06 mM	-	Berger and Evans, 1991	no
K_M ATP of PfkA	0.018 mM	-	Ogawa et al., 2007	no
K_M ATP of PfkA	0.057 mM	-	Auzat et al., 1994	no
K_M ATP of PfkA	0.041 mM	-	Hellinga and Evans, 1987	no
K_M ADP of PfkA	0.025 mM	0.5 (chosen)	Blangy and Monod, 1968	yes
K_D T PEP of PfkA	0.75 mM	1.0 (chosen)	Blangy and Monod, 1968	no
K_I PEP of PfkA	1.953 mM	-	Ogawa et al., 2007	no
K_I PEP of PfkA	0.30 mM	0.01 mM	Paricharttanakul et al., 2005	no
K_I ADP of PfkA	0.048 mM	0.002 mM	Paricharttanakul et al., 2005	no

PPP - FBA3

Reaction equation

$1\ S17BP \rightarrow 1\ E4P + 1\ DHAP$

sedoheptulose 1,7-bisphosphate = erythrose 4-phosphate + dihydroxyacetone phosphate

BIGG: -1 s17bp_c +1 e4p_c +1 dhap_c

Enzymes and genes

EcoCyc and UniProt are the same as FbaA

FBA3 (sedoheptulose 1,7-bisphosphate D-glyceraldehyde-3-phosphate-lyase) is a promiscuous activity of FbaA. This activity was discovered when the TALA reaction was blocked by deletion of talA and talB, and by feeding either xylose or gluconate as substrate (Nakahigashi et al., 2009). The activity only occurs when S7P accumulates in the cell (e.g. blocking of the TALA reaction) and would thus not likely occur in the wild type and is better to leave out of the model (Riemer et al., 2013). This promiscuous activity is slower than the native activity, so lower affinity for S17BP as substrate compared to FDP. FbaA is a homodimeric enzyme. A Δ*fbaA* mutant is viable, but has a heat-sensitive defect in rRNA transcription. Zn^{2+} is a cofactor of FbaA. (sedoheptulose biphosphate bypass)

Regulation

Gene regulation: Fructofuranose 1-phosphate binds to Cra (DNA-binding transcription factor) to block inhibition, while Cra inhibits transcription initiation of *fbaA*, thus an increase in fructofuranose 1-phosphate increases transcription of *fbaA*. cAMP binds to CRP (DNA-binding transcription factor) to enable activation and CRP activates transcription initiation of *fbaA*.

Activators: Activation of FbaA by NH_4 (ammonium) and K^+ (Blom et al., 1996).

Inhibitors: Inhibition of FbaA by Ni^{2+} (nickel; Macomber et al., 2011) and product inhibition of FbaA by DHAP (competitive; dihydroxyacetone phosphate; Plater et al., 1999) and G3P (uncompetitive). Competitive inhibition of FbaA by 2-phosphoglycolate (2PGly; Qamar et al., 1996).

Reaction and enzyme constants

Parameter	Value	Uncertainty	Reference	In current model
Reversible	yes	-	-	-
k_{cat}	0.052 1/s	-	Nakahara et al., 2003; yeast (calculated from specific activity)	no
k_{appt}^{max} x2	17.9 x2 = 35.8 1/s	0.2 (chosen)	Calculated from experimental fluxes and protein concentrations (WT)	yes
Formation energy S17BP	-2257.1 kJ/mol	4.0 kJ/mol	eQuilibrator	yes
Formation energy E4P	-1156.0 kJ/mol	3.4 kJ/mol	eQuilibrator	yes
Formation energy DHAP	-1097.2 kJ/mol	1.1 kJ/mol	eQuilibrator	yes
K_M S17BP	0.23 mM	0.5 (chosen)	Flechner et al., 1999; *Cyanophora paradoxa*	yes
K_M S17BP	0.625 mM	-	Nakahara et al., 2003; yeast	no
K_M E4P	0.088 mM	1.5 (chosen)	Babul et al., 1993 (maybe the same as DHAP, the other product)	yes
K_M DHAP of FbaA	0.03 mM	0.002 mM	Zgiby et al., 2002 (WT)	no
K_M DHAP of FbaA	0.088 mM	0.5 (chosen)	Babul et al., 1993	yes
K_I DHAP of FbaA	0.13 mM	0.011 mM	Plater et al., 1999	no
K_I G3P of FbaA	0.6 mM	-	Babul et al., 1993	no
K_I 2PGly of FbaA	0.009 mM	0.0012 mM	Qamar et al., 1996	no

Shikimate - DDPA

Reaction equation

$$1\ PEP + 1\ E4P + 1\ H_2O \rightarrow 1\ 2DDA7P + 1\ Pi$$

phosphoenolpyruvate + erythrose 4-phosphate + H_2O = 2-dehydro-3-deoxyarabino-heptulosonate 7-phosphate + phosphate

BIGG: -1 pep_c -1 e4p_c (-1 h2o_c) +1 2dda7p_c +1 pi_c

Enzymes and genes

EcoCyc Tyr, EcoCyc Phe, EcoCyc Trp and UniProt Tyr, UniProt Phe, UniProt Trp

The DDPA (3-deoxy-7-phosphoheptulonate synthase) reaction consists of three isozymes: DDPA Tyr-sensitive encoded by the *aroF* gene, DDPA Phe-sensitive encoded by the *aroG* gene, and DDPA Trp-sensitive encoded by the *aroH* gene. DDPA Tyr is an homodimeric enzyme, DDPA Phe is an homotetrameric enzyme, DDPA Trp is an homodimeric enzyme, and all isozymes are located in the cytosol. There is a high degree of sequence identity (41%) between the three isozymes and the polypeptides are nearly identical in size. DDPA Phe makes up about 80% of the total DDPA activity, DDPA Tyr makes up about 20%, and DDPA Trp makes up about 1% (Tribe et al., 1976). All three isozymes are metalloenzymes and require a divalent metal for catalysis and/or structural integrity: DDPA Tyr can use several metal ions, DDPA Phe uses Fe^{2+} mostly, and DDPA Trp is activated by Fe^{2+}. Certain mutations in all three isozymes lead to insensitivity towards the corresponding aromatic amino acid. The role of metal ion in DDPA is to position the amino acids with the appropriate geometry required to coordinate and activate the water molecule; the rate constant varies with the bound metal ion (Furdui et al., 2004).

Regulation

Gene regulation: SoxR (DNA-binding transcription factor) activates transcription initiation of *aroF,* Nac (DNA-binding transcription factor) inhibits transcription initiation of *aroF*, and L-tyrosine binds to TyrR (DNA-binding transcription factor) to enable inhibition and TyrR inhibits transcription initiation of *aroF*. L-leucine binds to Lrp (DNA-binding transcription factor) to block either activation or inhibition of transcription initiation of *aroG* by Lrp. Phosphate binds to CpxR (DNA-binding transcription factor) to enable activation and CpxR activates transcription initiations of *aroG*, while L-phenylalanine binds to TyrR (DNA-binding transcription factor) to enable inhibition and TyrR inhibits transcription initiation of *aroG*. Competitive inhibition of L-tyrosine and L-phenylalanine binding to TyrR. L-tryptophan binds to TrpR (DNA-binding transcription factor) to enable inhibition and TrpR inhibits transcription initiation of *aroH*.

Activators: none or unknown.

Inhibitors: Allosteric (potential) inhibition of DDPA Tyr by L-tyrosine, noncompetitive inhibition of DDPA Tyr by phosphate and competitive for PEP (product) inhibition of DDPA Tyr by 2DDA7P (2-dehydro-3-deoxyarabino-heptulosonate 7-phosphate) (Schoner and Herrmann, 1976). Allosteric inhibition of DDPA Phe by L-phenylalanine (McCandliss et al., 1978) and inhibition of DDPA Phe by L-alanine and L-dihydrophenylalanine (McCandliss et al., 1978). Competitive inhibition of DDPA Phe by 2,3-bisphosphoglycerate, 2-phosphoglycerate (2PG; glycerate 2-phosphate), 3-methylphosphoenolpyruvate, and 3-propylphosphoenolpyruvate (Simpson and Davidson, 1976). (Noncompetitive, possibly allosteric) inhibition of DDPA Trp by L-tryptophan (Ray and Bauerle, 1991; Camakaris and Pittard, 1974) and DDPA Trp follows non-Michaelis-Menten kinetics (Akowski and Bauerle, 1997).

Reaction and enzyme constants

Parameter	Value	Uncertainty	Reference	In current model
Reversible	no	-	-	-
k_{cat} DDPA Tyr	60.3 1/s	-	Schoner and Herrmann, 1976 (based on specific activity)	no
k_{cat} DDPA Tyr	29.5 1/s	0.2 (chosen)	Ramilo and Evans, 1997	yes
k_{cat} DDPA Phe	32.0 1/s	-	Sundaram et al., 1998	no
k_{cat} DDPA Phe	62.3 1/s	0.5 1/s; 0.2 (chosen)	Xu et al., 2004	yes
k_{cat} DDPA Phe	71.0 1/s	-	Williamson et al., 2005	no
k_{cat} DDPA Trp	20.6 1/s	1.1 1/s; 0.2 (chosen)	Akowski and Bauerle, 1997	yes
Formation energy PEP	-1205.1 kJ/mol	1.6 kJ/mol	eQuilibrator	no
Formation energy E4P	-1156.0 kJ/mol	3.4 kJ/mol	eQuilibrator	no
Formation energy 2DDA7P	-1513.8 kJ/mol	2.7 kJ/mol	eQuilibrator	no
Formation energy Pi	-1072.6 kJ/mol	1.5 kJ/mol	eQuilibrator	no
K_M PEP of DDPA Tyr	0.00585 mM	-	Schoner and Herrmann, 1976	no
K_M PEP of DDPA Tyr	0.013 mM	0.2 (chosen)	Ramilo and Evans, 1997	yes
K_M E4P of DDPA Tyr	0.0965 mM	-	Schoner and Herrmann, 1976	no
K_M E4P of DDPA Tyr	0.0814 mM	0.2 (chosen)	Ramilo and Evans, 1997	yes
K_M PEP of DDPA Phe	0.08 mM	0.04 mM	Simpson and Davidson, 1976	no
K_M PEP of DDPA Phe	0.009 mM	-	Sundaram et al., 1998	no
K_M PEP of DDPA Phe	0.035 mM	0.004 mM; 0.5 (chosen)	Xu et al., 2004	yes
K_M E4P of DDPA Phe	0.9 mM	0.3 mM	Simpson and Davidson, 1976	no
K_M E4P of DDPA Phe	0.086 mM	-	Sundaram et al., 1998	no
K_M E4P of DDPA Phe	0.25 mM	0.052 mM; 0.2 (chosen)	Xu et al., 2004	yes
K_M E4P of DDPA Phe	0.021 mM	-	Williamson et al., 2005	no
K_M ($S_{0.5}$) PEP of DDPA Trp	0.0053 mM	0.2 (chosen)	Akowski and Bauerle, 1997	yes
K_M E4P of DDPA Trp	0.076 mM	0.005 mM	Camakaris and Pittard, 1974	no
K_M ($S_{0.5}$) E4P of DDPA Trp	0.035 mM	0.2 (chosen)	Akowski and Bauerle, 1997	yes
K_M 2DDA7P	0.01 mM	2.0 (chosen)	Picked based on previous simulation	no
K_M Pi	1.0 mM	1.5 (chosen)	Picked (weakly informative)	no
K_I (K_D T) Tyr of DDPA Tyr	0.009 mM	0.2 (chosen)	Ramilo and Evans, 1997	yes
K_I Tyr of DDPA Tyr	0.082 mM	-	McCandliss et al., 1978	no
K_I (K_D T) PHE of DDPA Phe	0.013 mM	0.2 (chosen)	McCandliss et al., 1978	yes
K_I 2PG of DDPA Phe	1.0 mM	0.1 mM	Simpson and Davidson, 1976	no
K_I (K_D T) Trp of DDPA Trp	0.0014 mM	0.2 (chosen)	Akowski and Bauerle, 1997	yes

Shikimate - DHQS

Reaction equation

$1\ 2DDA7P \rightarrow 1\ 3DHQ + 1\ Pi$

2-dehydro-3-deoxyarabino-heptulosonate 7-phosphate = 3-dehydroquinate + phosphate

BIGG: -1 2dda7p_c +1 3dhq_c +1 pi_c

Enzymes and genes

EcoCyc and UniProt

DHQS (3-dehydroquinate synthase) is a monomeric enzyme encoded by the *aroB* gene and is located in the cytosol. DHQS requires multiple cofactors: NAD^+ and Zn^{2+} or Co^{2+}. Co^{2+} as cofactor results in higher specific activity, Zn^{2+} is more readily available in nature.

Regulation

Gene regulation: Glutarate binds to GlaR (DNA-binding transcription factor) to block inhibition, while GlaR inhibits transcription initiation of *aroB*, thus an increase in glutarate increases transcription of *aroB*. It is noteworthy that *aroB* is less regulated than the other genes in the shikimate pathway and the *aroB* expression is not repressed by chorismate, any of the aromatic amino acids or by the transcription factors TrpR and TyrR (Tribe et al., 1976).

Activators: none or unknown (apart from the cofactors; Bender et al., 1989).

Inhibitors: Competitive inhibition by 2DDA7P (2-dehydro-3-deoxyarabino-heptulosonate 7-phosphate; substrate inhibition) and variants of 2DDA7P (Myrvold et al., 1989).

Reaction and enzyme constants

Parameter	Value	Uncertainty	Reference	In current model
Reversible	no	-	-	-
k_{cat}	72.9 or 137.5 1/s	0.2 (chosen)	Bender et al., 1989 (calculated for Zn^{2+})	yes
k_{cat}	16.0 1/s	0.2 1/s; 0.2 (chosen)	Negron and Parker, 2011	no
Formation energy 2DDA7P	-1513.8 kJ/mol	2.7 kJ/mol	eQuilibrator	no
Formation energy 3DHQ	-608.7 kJ/mol	12.1 kJ/mol	eQuilibrator	no
Formation energy Pi	-1072.6 kJ/mol	1.5 kJ/mol	eQuilibrator	no
K_M 2DDA7P	0.018 mM	-	Myrvold et al., 1989	no
K_M 2DDA7P	0.033 mM	-	Maitra and Sprinson, 1978	no
K_M 2DDA7P	0.0055 mM	0.2 (chosen)	Bender et al., 1989	yes
K_M 2DDA7P	0.0057 mM	0.5 (chosen)	Negron and Parker, 2011	no
K_M 3DHQ	0.002 mM	2.0 (chosen)	Picked based on previous simulation	no
K_M Pi	1.0 mM	1.5 (chosen)	Picked (weakly informative)	no
K_M NAD	80 nM	-	Bender et al., 1989	no

Shikimate - DHQTi

Reaction equation

$$1\ 3DHQ \rightarrow 1\ 3DHSK + 1\ H_2O$$

3-dehydroquinate = 3-dehydroshikimate + H_2O

BIGG: -1 3dhq_c +1 3dhsk_c (+1 h2o_c)

Enzymes and genes

EcoCyc and UniProt

DQDH (3-dehydroquinate dehydratase) is an homodimeric enzyme encoded by the *aroD* gene and is located in the cytosol.

Regulation

Gene regulation: Nac (DNA-binding transcription factor) inhibits transcription initiation of *aroD*.

Activators: none or unknown.

Inhibitors: Competitive inhibition by acetate, succinate, tartrate, and chloride; inhibition by diethylpyrocarbonate, and sodium borohydride (Chaudhuri et al., 1986).

Reaction and enzyme constants

Parameter	Value	Uncertainty	Reference	In current model
Reversible	yes	-	-	-
k_{cat}	135.0 1/s	-	Kleanthous et al., 1992	no
k_{cat}	142.0 1/s	2 1/s; 0.2 (chosen)	Leech et al., 1995	yes
k_{cat}	29.53 1/s	-	Liu et al., 2015	no
Formation energy 3DHQ	-608.7 kJ/mol	12.1 kJ/mol	eQuilibrator	no
Formation energy 3DHSK	-463.0 kJ/mol	12.2 kJ/mol	eQuilibrator	no
K_M 3DHQ	0.018 mM	-	Chaudhuri et al., 1986	no
K_M 3DHQ	0.073 mM	-	Chaudhuri et al., 1986 (different buffer)	no
K_M 3DHQ	0.44 mM	-	Mitsuhashi and Davis, 1954	no
K_M 3DHQ	0.016 mM	-	Kleanthous et al., 1992	no
K_M 3DHQ	0.017 mM	0.0009 mM; 0.2 (chosen)	Leech et al., 1995	yes
K_M 3DHQ	0.188 mM	-	Liu et al., 2015	no
K_M 3DHSK	0.004 mM	0.2 (chosen)	Picked based on previous simulation	yes
K_I acetate	102 mM	-	Chaudhuri et al., 1986	no
K_I succinate	74 mM	-	Chaudhuri et al., 1986	no
K_I chloride	17 mM	-	Chaudhuri et al., 1986	no
K_I tartrate	21 mM	-	Chaudhuri et al., 1986	no

Shikimate - SHK3Dr

Reaction equation

$$1\ 3DHSK + 1\ NADPH \rightarrow 1\ SKM + 1\ NADP^{+}$$

3-dehydroshikimate + NADPH = shikimate + $NADP^{+}$

BIGG: -1 3dhsk_c -1 nadph_c +1 skm_c +1 nadp_c

Enzymes and genes

EcoCyc and UniProt

SHK3Dr (shikimate dehydrogenase) is a monomeric enzyme encoded by the *aroE* gene and is located in the cytosol. Another SHK3Dr homodimeric enzyme is encoded by the *ydiB* gene and kinetic properties are available (EcoCyc and UniProt). The SHK3Dr from *aroE* is $NADP^{+}$-specific and has much higher catalytic efficiency than the SHK3Dr from *ydiB*, which has broader substrate specificity and can use either $NADP^{+}$ or NAD^{+} as a co-substrate (Michel et al., 2003). Mutant data and the results from metabolic engineering experiments strongly suggest that the SHK3Dr from *ydiB* is unable to replace the SHK3Dr from *aroE* under normal physiological conditions. An Δ*aroE* mutant is viable, but accumulates 3-dehydroshikimate in minimal medium and is able to grow on media supplemented with shikimate, phenylalanine, and tyrosine. In the "reverse" direction, AroE appears to be able to further dehydrogenate 3-dehydroshikimate to 3,5-dehydroshikimate, which can spontaneously convert to gallic acid (Muir et al., 2011). The physiological relevance is unclear.

Regulation

Gene regulation: Phosphate binds to ZraR (DNA-binding transcription factor) to enable activation and ZraR activates transcription initiation of *aroE*. Nac (DNA-binding transcription factor) inhibits transcription initiation of *aroE*.

Activators: none or unknown.

Inhibitors: (Linear) mixed inhibition by shikimate (Dell and Frost, 1993).

Reaction and enzyme constants

Parameter	Value	Uncertainty	Reference	In current model
Reversible	no	-	-	-
k_{cat}	236.7 1/s	0.2 (chosen)	Michel et al., 2003	no
k_{cat}	8750.0 1/s	-	Muir et al., 2011	no
k_{cat}	190.0 1/s	5.0 1/s; 0.2 (chosen)	García-Guevara et al., 2017	no
k_{cat}	178.0 1/s	7.0 1/s; 0.2 (chosen)	Noble et al., 2006	yes
Formation energy 3DHSK	-463.0 kJ/mol	12.2 kJ/mol	eQuilibrator	no
Formation energy SKM	-403.4 kJ/mol	12.3 kJ/mol	eQuilibrator	no
Formation energy $NADP^{+}$	-2033.6 kJ/mol	13.4 kJ/mol	eQuilibrator	no
Formation energy NADPH	-1967.4 kJ/mol	13.4 kJ/mol	eQuilibrator	no
K_M 3DHSK	0.11 mM	0.5 (chosen)	Draths et al., 1999	no
K_M 3DHSK	0.11 mM	0.017 mM; 0.2 (chosen)	Noble et al., 2006	yes
K_M SKM	0.065 mM	0.5 (chosen)	Michel et al., 2003	no
K_M SKM	0.102 mM	-	Muir et al., 2011	no
K_M SKM	0.13 mM	0.013 mM	García-Guevara et al., 2017	no
K_M SKM	0.095 mM	0.010 mM	Noble et al., 2006	no
K_M $NADP^{+}$	0.056 mM	-	Michel et al., 2003	no
K_M $NADP^{+}$	0.347 mM	-	Muir et al., 2011	no
K_M $NADP^{+}$	0.058 mM	0.007 mM; 0.5 (chosen)	García-Guevara et al., 2017	no
K_M $NADP^{+}$	0.011 mM	0.0025 mM	Noble et al., 2006	no
K_M NADPH	0.0126 mM	0.0022 mM; 0.2 (chosen)	Noble et al., 2006	yes
K_I SKM	0.16 mM	-	Dell and Frost, 1993	no

Shikimate - SHKK

Reaction equation

$1\ SKM + 1\ ATP \rightarrow 1\ SKM5P + 1\ ADP$

shikimate + ATP = shikimate 5-phosphate + ADP

BIGG: -1 skm_c -1 atp_c +1 skm5p_c +1 adp_c

Enzymes and genes

EcoCyc SHKK I, EcoCyc SHKK II and UniProt SHKK I, UniProt SHKK II

The SHKK (shikimate kinase) reaction consists of two isozymes: SHKK I encoded by *aroK* and SHKK II encoded by *aroL*. Both enzymes are present in the cytosol, however SHKK I has approximately 100-fold lower affinity for shikimate (DeFeyter and Pittard, 1986) and a three-fold lower specific activity than SHKK II (Ding et al., 2016), so SHKK II is the major isozyme. SHKK I is relatively easy to isolate and is expressed constitutively compared to aromatic amino acid biosynthesis, so possibly other, still unknown, function. SHKK I and II are both monomeric enzymes which require Mg^{2+} as a cofactor. A double mutant Δ*aroK*Δ*aroL* cannot grow on minimal medium, even when supplemented with shikimate, L-phenylalanine and L-tyrosine.

Regulation

Gene regulation: L-arginine binds to ArgR (DNA-binding transcription factor) to enable inhibition and ArgR inhibits transcription initiation of *aroK*, so no gene regulation by aromatic amino acids. L-tyrosine binds to TyrR (DNA-binding transcription factor) to enable inhibition and TyrR inhibits transcription initiation of *aroL* and L-tryptophan binds to TrpR (DNA-binding transcription factor) to enable inhibition and TrpR inhibits transcription initiation of *aroL*.

Activators: None or unknown.

Inhibitors: Substrate inhibition of SHKK II by shikimate (DeFeyter and Pittard, 1986).

Reaction and enzyme constants

Parameter	Value	Uncertainty	Reference	In current model
Reversible	no	-	-	-
k_{cat} SHKK I	6.0 1/s	-	Ding et al., 2016 (based on specific activity)	no
k_{cat} SHKK II	21.8 1/s	-	Ding et al., 2016 (based on specific activity)	no
k_{cat} SHKK II	32.0 1/s	0.2 (chosen)	DeFeyter and Pittard, 1986 (based on specific activity)	yes
Formation energy SKM	-403.4 kJ/mol	12.3 kJ/mol	eQuilibrator	no
Formation energy SKM5P	-1304.5 kJ/mol	11.2 kJ/mol	eQuilibrator	no
Formation energy ATP	-2280.7 kJ/mol	2.9 kJ/mol	eQuilibrator	no
Formation energy ADP	-1405.9 kJ/mol	2.4 kJ/mol	eQuilibrator	no
K_M SKM of SHKK I	20 mM	1.5 (chosen)	DeFeyter and Pittard, 1986	no
K_M SKM of SHKK I	0.4 mM	-	Durante-Rodríguez et al., 2013	no
K_M SKM5P of SHKK I	-	-	-	no
K_M ATP of SHKK I	-	-	-	no
K_M ADP of SHKK I	-	-	-	no
K_M SKM of SHKK II	0.2 mM	0.2 (chosen)	DeFeyter and Pittard, 1986	yes
K_M SKM5P of SHKK II	0.01 mM	2.0 (chosen)	Based on simulations	no
K_M ATP of SHKK II	0.16 mM	0.2 (chosen)	DeFeyter and Pittard, 1986	yes
K_M ADP of SHKK II	0.02 mM	1.5 (chosen)	Based on other Km values	no

Shikimate - PSCVT

Reaction equation

$1\,SKM5P + 1\,PEP \rightarrow 1\,3PSME + 1\,Pi$

shikimate 5-phophate + phosphoenolpyruvate = 5-O-(1-Carboxyvinyl)-3-phosphoshikimate + phosphate

BIGG: -1 skm5p_c -1 pep_c +1 3psme_c +1 pi_c

Enzymes and genes

EcoCyc and UniProt

PSCVT (3-phosphoshikimate 1-carboxyvinyltransferase or EPSP synthase) is a monomeric enzyme encoded by the *aroA* gene and is located in the cytosol. A Δ*aroA* mutants are auxotrophic for the aromatic amino acids and are unable to grow on minimal media.

Regulation

Gene regulation: L-leucine binds to Lrp (DNA-binding transcription factor) to block either inhibition or activation and Lrp inhibits or activates transcription initiation of *aroA*, depending on other factors. cAMP binds to CRP (DNA-binding transcription factor) to enable inhibition and the CRP-cAMP complex inhibits transcription initiation of *aroA*.

Activators: Activation by PEP (phosphoenolpyruvate; Gruys et al., 1992).

Inhibitors: Competitive inhibition of PEP by glyphosate (Duncan et al., 1984), inhibition by pyruvate (Huynh, 1992) and 3-bromopyruvate (Huynh, 1991) and product inhibition by 5-O-(1-Carboxyvinyl)-3-phosphoshikimate (3PSME or EPSP; Duncan et al., 1984).

Reaction and enzyme constants

Parameter	Value	Uncertainty	Reference	In current model
Reversible	yes	-	-	-
k_{cat}	56.7 1/s	0.8 1/s	Gruys et al., 1992	no
k_{cat}	17.2 1/s	-	Duncan et al., 1984 (based on specific activity)	no
k_{cat}	41.2 1/s	-	Huynh, 1987 (based on specific activity)	no
k_{cat}	50.6 1/s	0.2 (chosen)	Lewendon and Coggins, 1987 (based on specific activity)	yes
k_{cat}	8.2 1/s	-	Shuttleworth and Evans, 1996 (based on specific activity)	no
k_{cat}	26.4 1/s	-	Haghani et al., 2008	no
k_{cat}	14.0 1/s	1 1/s	Berti and Chindemi, 2009	no
k_{cat}	40.8 1/s	0.8 1/s	Healy-Fried et al., 2007 (based on specific activity)	no
k_{cat}	30.5 1/s	-	Eschenburg et al., 2002 (based on specific activity)	no
k_{cat}	46.6 1/s	0.8 1/s	Funke et al., 2009 (based on specific activity)	no
k_{cat}	43.3 1/s	2.5 1/s	Priestman et al., 2005 (based on specific activity)	no
Formation energy SKM5P	-1304.5 kJ/mol	11.2 kJ/mol	eQuilibrator	no
Formation energy PEP	-1205.1 kJ/mol	1.6 kJ/mol	eQuilibrator	no
Formation energy 3PSME	-1453.5 kJ/mol	11.8 kJ/mol	eQuilibrator	no
Formation energy Pi	-1072.6 kJ/mol	1.5 kJ/mol	eQuilibrator	no
K_M SKM5P	0.0032 mM	0.0002 mM	Gruys et al., 1992	no
K_M SKM5P	0.0025 mM	-	Duncan et al., 1984	no
K_M SKM5P	0.020 mM	-	Huynh, 1987	no
K_M SKM5P	0.0025 mM	0.2 (chosen)	Lewendon and Coggins, 1987	yes
K_M SKM5P	0.135 mM	-	Shuttleworth and Evans, 1996	no
K_M SKM5P	0.14 mM	0.02 mM	Haghani et al., 2008	no
K_M SKM5P	0.060 mM	0.006 mM	Healy-Fried et al., 2007	no
K_M SKM5P	0.008 mM	0.004 mM	Shuttleworth et al., 1999	no
K_M SKM5P	0.12 mM	-	Eschenburg et al., 2002	no
K_M SKM5P	0.048 mM	0.005 mM	Funke et al., 2009	no
K_M SKM5P	0.09 mM	0.005 mM	Priestman et al., 2005	no
K_M PEP	0.021 mM	0.001 mM	Gruys et al., 1992	no
K_M PEP	0.016 mM	-	Duncan et al., 1984	no
K_M PEP	0.025 mM	-	Huynh, 1987	no
K_M PEP	0.022 mM	0.006 mM	He et al., 2003	no
K_M PEP	22.5 mM	6.32 mM	He et al., 2001	no
K_M PEP	0.016 mM	0.2 (chosen)	Lewendon and Coggins, 1987	yes
K_M PEP	0.1 mM	-	Shuttleworth and Evans, 1996	no
K_M PEP	0.1 mM	-	Haghani et al., 2008	no
K_M PEP	0.16 mM	0.02 mM	Haghani et al., 2008	no
K_M PEP	0.060 mM	0.006 mM	Healy-Fried et al., 2007	no
K_M PEP	0.013 mM	0.004 mM	Shuttleworth et al., 1999	no
K_M PEP	0.088 mM	-	Eschenburg et al., 2002	no
K_M PEP	0.045 mM	0.005 mM	Funke et al., 2009	no

K_M PEP	0.10 mM	0.004 mM	Priestman et al., 2005	no
K_M 3PSME	0.003 mM	-	Duncan et al., 1984	no
K_M 3PSME	0.003 mM	0.2 (chosen)	Lewendon and Coggins, 1987	yes
K_M 3PSME	0.011 mM	-	Shuttleworth and Evans, 1996	no
K_M 3PSME	0.010 mM	0.005 mM	Shuttleworth et al., 1999	no
K_M Pi	2.5 mM	-	Duncan et al., 1984	no
K_M Pi	2.5 mM	0.2 (chosen)	Lewendon and Coggins, 1987	yes
K_M Pi	4.6 mM	-	Shuttleworth and Evans, 1996	no
K_M Pi	5 mM	2 mM	Shuttleworth et al., 1999	no
K_I glyphosate	0.009 mM	-	Duncan et al., 1984	no
K_I glyphosate	0.0015 mM	0.0005 mM	He et al., 2003	no
K_I glyphosate	0.96 mM	0.4 mM	He et al., 2001	no
K_I glyphosate	0.009 mM	-	Lewendon and Coggins, 1987	no
K_I glyphosate	0.0012 mM	-	Shuttleworth and Evans, 1996	no
K_I glyphosate	0.0004 mM	-	Healy-Fried et al., 2007	no
K_I glyphosate	0.00013 mM	-	Shuttleworth et al., 1999	no
K_I glyphosate	0.0003 mM	-	Funke et al., 2009	no

Shikimate - CHORS

Reaction equation

$1\ 3PSME \rightarrow 1\ CHOR + 1\ Pi$

5-O-(1-Carboxyvinyl)-3-phosphoshikimate + phosphate = chorismate + phosphate

BIGG: -1 3psme_c +1 chor_c +1 pi_c

Enzymes and genes

EcoCyc and UniProt

CHORS (chorismate synthase) is a homotetrameric enzyme encoded by the *aroC* gene and is located in the cytosol. The flavin $FMNH_2$ is required as cofactor for CHORS, $FADH_2$ works as well but $FMNH_2$ is favoured. The CHORS enzyme is inactive under aerobic conditions, because it is oxygen sensitive. A Δ*aroC* mutant is unable to grow in minimal medium.

Regulation

Gene regulation: none or unknown.

Activators: none or unknown. Structural (conformational) changes upon flavin and substrate binding (Macheroux et al., 1998). "The line through the data points assumes dissociation of the active tetramer to two inactive dimers (K_d = 0.25 nM) on dilution" (Ramjee et al., 1994)

Inhibitors: Competitive inhibition of 3PSME by 6-fluoro-3PSME variants (Osborne et al., 2000).

Reaction and enzyme constants

Parameter	Value	Uncertainty	Reference	In current model
Reversible	no	-	-	-
k_{cat}	16.5 1/s	0.2 (chosen)	Ramjee et al., 1994	no
k_{cat}	29.0 1/s	3 1/s; 0.2 (chosen)	Bornemann et al., 1996	no
k_{appt}^{max} x2	30.3 x 2 = 60.6 1/s	0.2 (chosen)	Calculated from experimental fluxes and protein concentrations (wt)	yes
Formation energy 3PSME	-1453.5 kJ/mol	11.8 kJ/mol	eQuilibrator	no
Formation energy CHOR	-443.6 kJ/mol	15.0 kJ/mol	eQuilibrator	no
Formation energy Pi	-1072.6 kJ/mol	1.5 kJ/mol	eQuilibrator	no
K_M 3PSME	0.0013 mM	0.0003 mM; 0.2 (chosen)	Ramjee et al., 1994	yes
K_M CHOR	0.01 mM	2.0 (chosen)	Based on simulations	no
K_M Pi	2.5 mM	1.5 (chosen)	Based on other Km values of PSCVT	no

Phe/Tyr - CHORM

Reaction equation

$1\ CHOR \rightarrow 1\ PPHN$

chorismate = prephenate

BIGG: -1 chor_c +1 pphn_c

Enzymes and genes

EcoCyc PheA, EcoCyc TyrA and UniProt PheA, UniProt TyrA

The CHORM (chorismate mutase) reaction consists of two (iso)enzymes: PheA encoded by the *pheA* gene and TyrA encoded by the *tyrA* gene. Both enzymes are homodimeric and are located in the cytosol. PheA is a fused (bifunctional) chorismate mutase/prephenate dehydratase and thus catalyses both reactions. The native enzyme is a dimer of identical subunits each containing a dehydratase active site, a mutase active site and a phenylalanine binding site. Prephenate, which is formed from chorismate, dissociates from the mutase site and equilibrates with the bulk medium before combining at the dehydratase site (Duggleby et al., 1978). TyrA is a fused (bifunctional) chorismate mutase/prephenate dehydrogenase and thus catalyses both reactions. The two catalytic activities of TyrA occur in separate portions of the protein. Specifically, the chorismate mutase activity requires the amino-terminal portion of the protein, and the prephenate dehydrogenase activity is in the carboxy-terminal portion of the protein. A mol of dimerised TyrA can bind roughly one mol of NAD^+, one mol of tyrosine, or one mol of prephenate. Both PheA and TyrA are common targets for metabolic engineering to increase titers of phenylalanine and tyrosine, respectively.

Regulation

Gene regulation: none or unknown for PheA. L-tyrosine binds to TyrR (DNA-binding transcription factor) to enable inhibition and the tyr-TyrR complex inhibits transcription initiation of *tyrA*. This complex is in turn inhibited by L-phenylalanine, thus an increase in L-phenylalanine increases transcription of *tyrA*. SoxS (DNA-binding transcription factors) activates transcription initiation of *tyrA*.

Activators: none or unknown.

Inhibitors: Allosteric inhibition of PheA by L-phenylalanine (Baldwin and Davidson, 1981; Dopheide et al., 1972) and competitive inhibition of PheA by citrate (Baldwin and Davidson, 1983) and prephenate (Gething and Davidson, 1977; Duggleby et al., 1978). Allosteric inhibition of TyrA by L-tyrosine (Hudson et al., 1983; Lütke-Eversloh and Stephanopoulos, 2005), competitive inhibition by prephenate to chorismate (Koch et al., 1972; Rood et al., 1982) and noncompetitive inhibition by citrate (Rood et al., 1982).

Reaction and enzyme constants

Parameter	Value	Uncertainty	Reference	In current model
Reversible	no	-	-	-
k_{cat} PheA	14.7 1/s	0.1 1/s; 0.2 (chosen)	Duggleby et al., 1978 (calculated from specific activity)	yes
k_{cat} PheA	34.7 1/s	0.2 (chosen)	Baldwin and Davidson, 1981 (calculated from specific activity)	no
k_{cat} PheA	40.0 1/s	-	Stewart et al., 1990 (calculated from specific activity; only CHORM)	no
k_{cat} PheA	72.0 1/s	-	Liu et al., 1996 (only CHORM)	no
k_{cat} PheA	41.4 1/s	4.8 1/s	Zhang et al., 1996 (only CHORM)	no
k_{cat} PheA	39.0 1/s	4.3 1/s	Zhang et al., 1998	no
k_{cat} PheA	34.2 1/s	1.4 1/s	Zhang et al., 2000	no
k_{cat} PheA	38.9 1/s	5.1 1/s	Lassila et al., 2005	no
k_{appt}^{max} x2 PheA	31.0 x 2 = 62.0 1/s	0.2 (chosen)	Calculated from experimental fluxes and protein concentrations (wt)	no
k_{cat} TyrA	71.0 1/s	-	Hudson et al., 1983	no
k_{cat} TyrA	27.0 1/s	0.7 1/s; 0.2 (chosen)	Christendat et al., 1998	yes
k_{appt}^{max} x2 TyrA	29.1 x2 = 58.2 1/s	0.2 (chosen)	Calculated from experimental fluxes and protein concentrations (Δ*sdhCB*)	no
Formation energy CHOR	-443.6 kJ/mol	15.0 kJ/mol	eQuilibrator	no
Formation energy PPHN	-492.2 kJ/mol	20.1 kJ/mol	eQuilibrator	no
K_M CHOR of PheA	0.045 mM	-	Dopheide et al., 1972	no
K_M CHOR of PheA	0.044 mM	0.009 mM	Gething and Davidson, 1977	no
K_M CHOR of PheA	0.024 mM	0.001 mM; 0.2 (chosen)	Duggleby et al., 1978	yes
K_M CHOR of PheA	0.031 mM	0.003 mM; 0.5 (chosen)	Baldwin and Davidson, 1983	no
K_M CHOR of PheA	0.29 mM	-	Stewart et al., 1990 (only CHORM)	no
K_M CHOR of PheA	0.296 mM	0.019 mM	Liu et al., 1996 (only CHORM)	no
K_M CHOR of PheA	0.3 mM	0.01 mM	Zhang et al., 1996 (only CHORM)	no
K_M CHOR of PheA	0.226 mM	0.025 mM	Zhang et al., 1998	no
K_M CHOR of PheA	0.127 mM	0.019 mM	Zhang et al., 2000	no
K_M CHOR of PheA	0.304 mM	0.052 mM	Lassila et al., 2005	no
K_M PPHN of PheA	-	-	-	-
K_M CHOR of TyrA	0.39 mM	-	Koch et al., 1971	no
K_M CHOR of TyrA	0.14 mM	0.01 mM	Rood et al., 1982	no
K_M CHOR of TyrA	0.092 mM	-	Hudson et al., 1983	no
K_M CHOR of TyrA	0.045 mM	0.007 mM; 0.2 (chosen)	Christendat et al., 1998	yes
K_M PPHN of TyrA	-	-	-	-
K_D T PHE of PheA	0.02013 mM	0.003 mM; 0.2 (chosen)	Zhang et al., 1998	yes
K_I PPHN of PheA	0.047 mM	0.008 mM	Gething and Davidson, 1977	no
K_I PPHN of PheA	0.031 mM	0.002 mM	Duggleby et al., 1978	no
K_I CIT of PheA	1.01 mM	0.09 mM	Baldwin and Davidson, 1983	no
K_D T TYR of TyrA	0.01 mM	1.0 (chosen)	Weakly informative	yes

Phenylalanine - PPNDH

Reaction equation

$$1\ PPHN \rightarrow 1\ PHPYR + 1\ CO_2 + 1\ H_2O$$

prephenate = phenylpyruvate + CO_2 + H_2O

BIGG: -1 pphn_c +1 phpyr_c (+1 co2_c +1 h2o_c)

Enzymes and genes

EcoCyc and UniProt

PPNHD (prephenate dehydratase) is a homodimeric enzyme encoded by the *pheA* gene and is located in the cytosol. PheA is a fused (bifunctional) chorismate mutase/prephenate dehydratase and thus catalyses both reactions. The native enzyme is a dimer of identical subunits each containing a dehydratase active site, a mutase active site and a phenylalanine binding site. Prephenate, which is formed from chorismate, dissociates from the mutase site and equilibrates with the bulk medium before combining at the dehydratase site (Duggleby et al., 1978).

Regulation

Gene regulation: none or unknown.

Activators: none or unknown.

Inhibitors: Allosteric inhibition by L-phenylalanine (Baldwin and Davidson, 1981; Dopheide et al., 1972), competitive inhibition by aconitate (Baldwin and Davidson, 1983) and inhibition by chorismate (Duggleby et al., 1978).

Reaction and enzyme constants

Parameter	Value	Uncertainty	Reference	In current model
Reversible	no	-	-	-
k_{cat}	12.0 1/s	0.1 1/s	Duggleby et al., 1978 (calculated from specific activity)	no
k_{cat}	21.3 1/s	0.2 (chosen)	Baldwin and Davidson, 1981 (calculated from specific activity)	yes
k_{cat}	26.2 1/s	1 1/s	Zhang et al., 1998	no
k_{cat}	32.2 1/s	1.6 1/s	Zhang et al., 2000	no
Formation energy PPHN	-492.2 kJ/mol	20.1 kJ/mol	eQuilibrator	no
Formation energy PHPYR	-20.0 kJ/mol	7.3 kJ/mol	eQuilibrator	no
Formation energy CO_2	-403.1 kJ/mol	5.7 kJ/mol	eQuilibrator	no
K_M PPHN	1.0 mM	-	Dopheide et al., 1972	no
K_M PPHN	0.47 mM	0.01 mM; 0.2 (chosen)	Duggleby et al., 1978	yes
K_M PPHN	0.549 mM	0.058 mM	Zhang et al., 1998	no
K_M PPHN	0.559 mM	0.078 mM	Zhang et al., 2000	no
K_M PHPYR	-	-	-	-
K_D T PHE of PheA	0.02013 mM	0.003 mM	Zhang et al., 1998	no
K_I CHOR	26.0 mM	10.4 mM	Duggleby et al., 1978	no
K_I PHPYR	4.6 mM	0.6 mM	Duggleby et al., 1978	no

Phenylalanine - PHETA1

Reaction equation

$1\ PHPYR + 1\ GLU \rightarrow 1\ PHE + 1\ AKG$

phenylpyruvate + L-glutamate = L-phenylalanine + 2-oxoglutarate

BIGG: -1 phe_L_c -1 akg_c +1 phpyr_c +1 glu_L_c

Enzymes and genes

EcoCyc TyrB, EcoCyc AspC, EcoCyc IlvE and UniProt TyrB, UniProt AspC, UniProt IlvE

The PHETA1 (phenylalanine transaminase) reaction consists of three isozymes: TyrB encoded by the *tyrB* gene, AspC encoded by the *aspC* gene and IlvE encoded by the *ilvE* gene. All isozymes are located in the cytosol. TyrB and AspC are homodimeric enzymes, while IlvE is a hexameric enzyme (a dimer of trimers). TyrB, AspC and IlvE are involved in catalyzing the third step of phenylalanine and tyrosine biosynthesis: all three can contribute to the synthesis of phenylalanine; only TyrB and AspC contribute to the biosynthesis of tyrosine. Under normal physiological conditions, TyrB is the primary enzyme contributing to the synthesis of tyrosine and phenylalanine. AspC contributes to their synthesis when substrate pools are large. The contribution of IlvE to phenylalanine biosynthesis was demonstrated in triple mutants of Escherichia coli K-12 that lacked all three aminotransferases and required both phenylalanine and tyrosine for growth. However, tyrB and aspC double mutants required only tyrosine for growth (Gelfand and Steinberg, 1977). TyrB is 1000-fold more active toward aromatic substrates than AspC (Hayashi et al., 1993). PLP (pyridoxal 5-phosphate) is a cofactor of TyrB, AspC (one per subunit) and IlvE. IlvE has very low activity for (tyrosine) and phenylalanine (Lee-Peng et al., 1979).

Regulation

Gene regulation: L-tyrosine binds to TyrR (DNA-binding transcription factor) to enable inhibition and the tyr-TyrR complex inhibits transcription initiation of *tyrB*. Fe^{2+} binds Fur (DNA-binding transcription factor) to enable inhibition of transcription initiation of *aspC* and FNR (DNA-binding transcription factors) activates or inhibits transcription initiation of *aspC*. L-leucine binds to Lrp (DNA-binding transcription factor) to block activation, while Lrp activates transcription initiation of *aspC*, thus an increase in L-leucine decreases transcription of *aspC*. L-leucine binds to Lrp (DNA-binding transcription factor) to enable inhibition and the Leu-Lrp complex inhibits transcription initiation of *ilvE*. ppGpp activates binding of RNA polymerase to the promoter of *ilvE* under conditions of stringent response.

Activators: none or unknown.

Inhibitors: Inhibition of TyrB by L-tyrosine (Collier and Kohlhaw, 1972), L-leucine (Powell and Morrison, 1978), and 3MOB (3-methyl-2-oxobutanoate; Vartak et al., 1991). Allosteric inhibition of AspC by 2-methylaspartate (Okamoto et al., 1994) and competitive inhibition of AspC by maleate (Miyahara et al., 1994). None or unknown for IlvE.

Reaction and enzyme constants

Parameter	Value	Uncertainty	Reference	In current model
Reversible	yes	-	-	-
k_{cat} TyrB	26.7 1/s	-	Mavrides and Orr, 1974 (calculated from specific activity)	no
k_{cat} TyrB	29.3 1/s	-	Gelfand and Steinberg, 1977 (calculated from specific activity)	no
k_{cat} TyrB	13.2 1/s	0.2 (chosen)	Powell and Morrison, 1978 (calculated from specific activity)	yes
k_{cat} TyrB	250.0 1/s	-	Hayashi et al., 1993	no
k_{cat} TyrB	520.0 1/s	-	Onuffer et al., 1995	no
k_{cat} TyrB	180.0 1/s	34 1/s	Luong and Kirsch, 1997	no
k_{cat} AspC	9.8 1/s	-	Mavrides and Orr, 1974 (calculated from specific activity)	no
k_{cat} AspC	52.1 1/s	-	Gelfand and Steinberg, 1977 (probably non-aromatic substrate; calculated from specific activity)	no
k_{cat} AspC	6.6 1/s	0.2 (chosen)	Powell and Morrison, 1978 (calculated from specific activity)	yes
k_{cat} AspC	13.8 1/s	2.3 1/s	Han et al., 2001	no
k_{cat} IlvE	0.8 1/s	0.2 (chosen)	Lee-Peng et al., 1979 (calculated from specific activity)	yes
Formation energy PHPYR	-20.0 kJ/mol	7.3 kJ/mol	eQuilibrator	no
Formation energy GLU	-361.7 kJ/mol	2.4 kJ/mol	eQuilibrator	no
Formation energy PHE	255.3 kJ/mol	5.7 kJ/mol	eQuilibrator	no
Formation energy AKG	-637.0 kJ/mol	2.6 kJ/mol	eQuilibrator	no
K_M PHPYR of TyrB	0.012 mM	-	Gelfand and Steinberg, 1977	no
K_M PHPYR of TyrB	0.056 mM	0.2 (chosen)	Powell and Morrison, 1978	yes
K_M GLU of TyrB	0.28 mM	0.2 (chosen)	Powell and Morrison, 1978	yes
K_M PHE of TyrB	0.333 mM	-	Mavrides and Orr, 1975	no
K_M PHE of TyrB	0.06 mM	0.2 (chosen)	Powell and Morrison, 1978	yes
K_M PHE of TyrB	0.26 mM	-	Hayashi et al., 1993	no
K_M PHE of TyrB	0.56 mM	0.03 mM	Luong and Kirsch, 1997	no
K_M AKG of TyrB	0.23 mM	0.2 (chosen)	Powell and Morrison, 1978	yes
K_M AKG of TyrB	1.7 mM	-	Hayashi et al., 1993	no
K_M AKG of TyrB	5 mM	1 mM	Luong and Kirsch, 1997	no
K_M PHPYR of AspC	3.9 mM	-	Gelfand and Steinberg, 1977	no
K_M PHPYR of AspC	0.65 mM	0.2 (chosen)	Powell and Morrison, 1978	yes
K_M GLU of AspC	0.9 mM	0.2 (chosen)	Powell and Morrison, 1978	yes
K_M GLU of AspC	15 mM		Yagi et al., 1985	no
K_M GLU of AspC	0.6 mM	-	Miyahara et al., 1994	no
K_M PHE of AspC	2.17 mM	-	Mavrides and Orr, 1975	no
K_M PHE of AspC	0.55 mM	0.2 (chosen)	Powell and Morrison, 1978	yes
K_M PHE of AspC	8 mM	3.6 mM	Han et al., 2001	no
K_M AKG of AspC	0.15 mM	0.2 (chosen)	Powell and Morrison, 1978	yes

K_M AKG of AspC	0.24 mM		Yagi et al., 1985	no
K_M AKG of AspC	0.47 mM	0.01 mM	Toney and Kirsch, 1991	no
K_M AKG of AspC	0.59 mM	0.05 mM	Deu et al., 2002	no
K_M PHPYR of IlvE	-	-	-	no
K_M GLU of IlvE	-	-	-	no
K_M PHE of IlvE	-	-	-	no
K_M AKG of IlvE	1.28 mM	-	Lee-Peng et al., 1979	no
K_I maleate	5.6 mM	0.5 mM	Toney and Kirsch, 1991	no

Tyrosine - PPND

Reaction equation

$$1\,PPHN + 1\,NAD^+ \rightarrow 1\,34HPP + 1\,CO_2 + 1\,NADH$$

prephenate + NAD^+ = 3-(4-hydroxyphenyl)pyruvate + CO_2 + NADH

BIGG: -1 pphn_c -1 nad_c +1 34hpp_c +1 nadh_c (+1 co2_c)

Enzymes and genes

EcoCyc and UniProt

PPND (prephenate dehydrogenase) is a homodimeric enzyme encoded by the *tyrA* gene and is located in the cytosol. TyrA is a fused (bifunctional) chorismate mutase/prephenate dehydrogenase and thus catalyses both reactions. The two catalytic activities of TyrA occur in separate portions of the protein. Specifically, the chorismate mutase activity requires the amino-terminal portion of the protein, and the prephenate dehydrogenase activity is in the carboxy-terminal portion of the protein. A mol of dimerised TyrA can bind roughly one mol of NAD^+, one mol of tyrosine, or one mol of prephenate. Both PheA and TyrA are common targets for metabolic engineering to increase titers of phenylalanine and tyrosine, respectively.

Regulation

Gene regulation: L-tyrosine binds to TyrR (DNA-binding transcription factor) to enable inhibition and the tyr-TyrR complex inhibits transcription initiation of *tyrA*. This complex is in turn inhibited by L-phenylalanine, thus an increase in L-phenylalanine increases transcription of *tyrA*. SoxS (DNA-binding transcription factors) activates transcription initiation of *tyrA*.

Activators: none or unknown.

Inhibitors: Competitive inhibition by L-tyrosine to prephenate (Hudson et al., 1983; Lütke-Eversloh and Stephanopoulos, 2005).

Reaction and enzyme constants

Parameter	Value	Uncertainty	Reference	In current model
Reversible	no	-	-	-
k_{cat} TyrA	71.0 1/s	-	Hudson et al., 1983	no
k_{cat} TyrA	27.0 1/s	1 1/s; 0.2 (chosen)	Christendat et al., 1998	yes
Formation energy PPHN	-492.2 kJ/mol	20.1 kJ/mol	eQuilibrator	no
Formation energy 34HPP	-183.2 kJ/mol	7.3 kJ/mol	eQuilibrator	no
Formation energy NAD^+	-1146.0 kJ/mol	13.1 kJ/mol	eQuilibrator	no
Formation energy NADH	-1079.8 kJ/mol	13.1 kJ/mol	eQuilibrator	no
Formation energy CO_2	-403.1 kJ/mol	5.7 kJ/mol	eQuilibrator	no
K_M PPHN	0.37 mM	-	Koch et al., 1971	no
K_M PPHN	0.13 mM	0.01 mM	Rood et al., 1982	no
K_M PPHN	0.044 mM	0.008 mM; 0.2 (chosen)	Christendat et al., 1998	yes
K_M 34HPP	-	-	-	-
K_M NAD^+	0.33 mM	-	Koch et al., 1971	no
K_M NAD^+	0.05 mM	-	Hudson et al., 1983	no
K_M NAD^+	0.103 mM	0.011 mM; 0.2 (chosen)	Christendat et al., 1998	yes
K_M NADH	-	-	-	-
K_I TYR	0.1 mM	-	Hudson et al., 1983	no

Tyrosine - TYRTA

Reaction equation

$1\ 34HPP + 1\ GLU \rightarrow 1\ TYR + 1\ AKG$

3-(4-hydroxyphenyl)pyruvate + L-glutamate = L-tyrosine + 2-oxoglutarate

BIGG: -1 tyr_L_c -1 akg_c +1 34hpp_c +1 glu_L_c

Enzymes and genes

EcoCyc TyrB, EcoCyc AspC and UniProt TyrB, UniProt AspC

The TYRTA (tyrosine aminotransferase) reaction consists in two isozymes: TyrB encoded by the *tyrB* gene and AspC encoded by the *aspC* gene. Both isozymes are homodimeric enzymes and located in the cytosol. TyrB, AspC and IlvE are involved in catalyzing the third step of phenylalanine and tyrosine biosynthesis: all three can contribute to the synthesis of phenylalanine; only TyrB and AspC contribute to the biosynthesis of tyrosine. Under normal physiological conditions, TyrB is the primary enzyme contributing to the synthesis of tyrosine and phenylalanine. AspC contributes to their synthesis when substrate pools are large. The contribution of IlvE to phenylalanine biosynthesis was demonstrated in triple mutants of Escherichia coli K-12 that lacked all three aminotransferases and required both phenylalanine and tyrosine for growth. However, tyrB and aspC double mutants required only tyrosine for growth (Gelfand and Steinberg, 1977). TyrB is 1000-fold more active toward aromatic substrates than AspC (Hayashi et al., 1993). PLP (pyridoxal 5-phosphate) is a cofactor of TyrB and AspC (one per subunit).

Regulation

Gene regulation: L-tyrosine binds to TyrR (DNA-binding transcription factor) to enable inhibition and the tyr-TyrR complex inhibits transcription initiation of *tyrB*. Fe^{2+} binds Fur (DNA-binding transcription factor) to enable inhibition of transcription initiation of *aspC* and FNR (DNA-binding transcription factors) activates or inhibits transcription initiation of *aspC*. L-leucine binds to Lrp (DNA-binding transcription factor) to block activation, while Lrp activates transcription initiation of *aspC*, thus an increase in L-leucine decreases transcription of *aspC*.

Activators: none or unknown.

Inhibitors: Inhibition of TyrB by L-tyrosine (Collier and Kohlhaw, 1972), L-leucine (Powell and Morrison, 1978), and 3MOB (3-methyl-2-oxobutanoate; Vartak et al., 1991). Allosteric inhibition of AspC by 2-methylaspartate (Okamoto et al., 1994) and competitive inhibition of AspC by maleate (Miyahara et al., 1994).

Reaction and enzyme constants

Parameter	Value	Uncertainty	Reference	In current model
Reversible	yes	-	-	-
k_{cat} TyrB	31.1 1/s	-	Mavrides and Orr, 1974 (calculated from specific activity)	no
k_{cat} TyrB	31.5 1/s	-	Gelfand and Steinberg, 1977 (calculated from specific activity)	no
k_{cat} TyrB	18.3 1/s	0.2 (chosen)	Powell and Morrison, 1978 (calculated from specific activity)	yes
k_{cat} TyrB	210.0 1/s	-	Hayashi et al., 1993	no
k_{cat} TyrB	660.0 1/s	-	Onuffer et al., 1995	no
k_{cat} AspC	10.1 1/s	-	Mavrides and Orr, 1974 (calculated from specific activity)	no
k_{cat} AspC	88.0 1/s	-	Gelfand and Steinberg, 1977 (probably non-aromatic substrate; calculated from specific activity)	no
k_{cat} AspC	6.6 1/s	0.2 (chosen)	Powell and Morrison, 1978 (calculated from specific activity)	yes
Formation energy 34HPP	-183.2 kJ/mol	7.3 kJ/mol	eQuilibrator	no
Formation energy GLU	-361.7 kJ/mol	2.4 kJ/mol	eQuilibrator	no
Formation energy TYR	91.7 kJ/mol	5.7 kJ/mol	eQuilibrator	no
Formation energy AKG	-637.0 kJ/mol	2.6 kJ/mol	eQuilibrator	no
K_M 34HPP of TyrB	0.013 mM	-	Gelfand and Steinberg, 1977	no
K_M 34HPP of TyrB	0.032 mM	0.2 (chosen)	Powell and Morrison, 1978	yes
K_M GLU of TyrB	0.28 mM	0.2 (chosen)	Powell and Morrison, 1978	yes
K_M TYR of TyrB	0.625 mM	-	Mavrides and Orr, 1975	no
K_M TYR of TyrB	0.042 mM	0.2 (chosen)	Powell and Morrison, 1978	yes
K_M TYR of TyrB	0.32 mM	-	Hayashi et al., 1993	no
K_M AKG of TyrB	0.23 mM	0.2 (chosen)	Powell and Morrison, 1978	yes
K_M AKG of TyrB	1.3 mM	-	Hayashi et al., 1993	no
K_M 34HPP of AspC	3.9 mM	-	Gelfand and Steinberg, 1977	no
K_M 34HPP of AspC	0.4 mM	0.2 (chosen)	Powell and Morrison, 1978	yes
K_M GLU of AspC	0.9 mM	0.2 (chosen)	Powell and Morrison, 1978	yes
K_M GLU of AspC	15 mM		Yagi et al., 1985	no
K_M GLU of AspC	0.6 mM	-	Miyahara et al., 1994	no
K_M TYR of AspC	1.43 mM	-	Mavrides and Orr, 1975	no
K_M TYR of AspC	0.45 mM	0.2 (chosen)	Powell and Morrison, 1978	yes
K_M AKG of AspC	0.15 mM	0.2 (chosen)	Powell and Morrison, 1978	yes
K_M AKG of AspC	0.24 mM		Yagi et al., 1985	no
K_M AKG of AspC	0.47 mM	0.01 mM	Toney and Kirsch, 1991	no
K_I maleate	5.6 mM	0.5 mM	Toney and Kirsch, 1991	no

Tryptophan - ANS

Reaction equation

$1\ CHOR + 1\ GLN \rightarrow 1\ ANTH + 1\ GLU + 1\ PYR$

chorismate + L-glutamine = anthranilate + L-glutamate + pyruvate

BIGG: -1 chor_c -1 gln_L_c +1 anth_c +1 glu_L_c + pyr_c

Enzymes and genes

EcoCyc and UniProt trpE, UniProt trpGD

The ANS (anthranilate synthase) reaction is catalysed by the TrpDE (TrpGDE) complex, a heterotetrameric enzyme (two trpD and two trpE subunits) located in the cytosol and encoded by the *trpD* and *trpE* genes. TrpE on its own can carry out an alternate version of this reaction, using ammonium sulfate rather than glutamine as an amino donor (Ito et al., 1969). However, TrpD dramatically increases the affinity of TrpE for glutamine over TrpE alone (Ito and Yanofsky, 1969). Mg^{2+} is preferred as cofactor, but Co^{2+} and Fe^{2+} work as well. Both mutants are not viable on minimal medium. The complex is more thermostable for both the ANS and ANPRT reactions, than the corresponding individual components. Drawing of subunits and conformational changes in Pabst et al., 1973.

Regulation

Gene regulation: L-tryptophan binds to TrpR (DNA-binding transcription factor) to enable inhibition and TrpR inhibits transcription initiation of *trpE* and *trpD*. L-tryptophanyl (tRNA for L-tryptophan promotes premature termination of transcription. Translation of TrpE and TrpD is coordinated via a specialized intercistronic sequence between trpE and trpD. If the latter portion of the trpE mRNA is not translated, trpD mRNA translation is markedly reduced.

Activators: None or unknown.

Inhibitors: Competitive inhibition of chorismate by L-tryptophan (Baker and Crawford, 1966, Pabst et al., 1973) and 7-mehtyl-L-tryptophan (Held and Smith, 1970). Noncompetitive inhibition with respect to ammonium sulfate or L-glutamine by L-tryptophan (Baker and Crawford, 1966), trpE alone is inhibited by L-tryptophan (competitive to chorismate; Ito et al., 1969).

Reaction and enzyme constants

Parameter	Value	Uncertainty	Reference	In current model
Reversible	no	-	-	-
k_{appt}^{max} x2	2.27 x2 = 4.54 1/s	0.2 (chosen)	Calculated from experimental fluxes and protein concentrations (ΔsdhCB)	yes
Formation energy CHOR	-443.6 kJ/mol	15.0 kJ/mol	eQuilibrator	no
Formation energy GLN	-104.9 kJ/mol	3.6 kJ/mol	eQuilibrator	no
Formation energy ANTH	-42.3 kJ/mol	5.7 kJ/mol	eQuilibrator	no
Formation energy GLU	-361.7 kJ/mol	2.4 kJ/mol	eQuilibrator	no
Formation energy PYR	-355.2 kJ/mol	1.5 kJ/mol	eQuilibrator	no
K_M CHOR	0.0055 mM	-	Ito and Yanofsky, 1969	no
K_M CHOR	0.005 mM	-	Pabst et al., 1973	no
K_M CHOR	0.0012 mM	0.2 (chosen)	Baker and Crawford, 1966	yes
K_M GLN	0.36 mM	0.2 (chosen)	Baker and Crawford, 1966	yes
K_M ANTH	-	-	-	-
K_M GLU	-	-	-	-
K_M PYR	-	-	-	-
K_M NH_4^+	39 mM	-	Ito and Yanofsky, 1969	no
K_M CHOR of trpE	0.03 mM	-	Ito and Yanofsky, 1969	no
K_M NH_4^+ of trpE	15 mM	-	Ito et al., 1969	no
K_M NH_4^+ of trpE	25 mM	-	Ito and Yanofsky, 1969	no
K_I (K_D T) TRP	0.001 mM	0.2(chosen)	Pabst et al., 1973	no

Tryptophan - ANPRT

Reaction equation

$1\ ANTH + 1\ PRPP \rightarrow 1\ PRAN + 1\ PPi$

anthranilate + 5-phosphoribose 1-diphosphate = N-(5-phosphoribosyl)anthranilate + diphosphate

BIGG: -1 anth_c -1 prpp_c +1 pran_c +1 ppi_c

Enzymes and genes

EcoCyc and UniProt

ANPRT (anthranilate phosphoribosyltransferase) is a monomeric (?) enzyme encoded by the *trpD* gene or the TrpDE complex (Jackson and Yanofsky, 1974) and is located in the cytosol. The phosphoribosyl transferase and anthranilate synthase contributing portions of TrpD are present in different portions of the protein. The anthranilate synthase reaction requires the amino-terminal portion of the protein, whereas the phosphoribosyltransferase reaction requires the carboxy-terminal region (Jackson and Yanofsky, 1974). A Δ*trpD* mutant is not viable in minimal medium.

Regulation

Gene regulation: L-tryptophan binds to TrpR (DNA-binding transcription factor) to enable inhibition and TrpR inhibits transcription initiation of *trpE* and *trpD*. L-tryptophanyl (tRNA for L-tryptophan promotes premature termination of transcription. Translation of TrpE and TrpD is coordinated via a specialized intercistronic sequence between trpE and trpD. If the latter portion of the trpE mRNA is not translated, trpD mRNA translation is markedly reduced.

Activators: None or unknown.

Inhibitors: Competitive inhibition of PRPP by L-tryptophan (Gonzalez and Somerville, 1986) or noncompetitive inhibition of PRPP by L-tryptophan (Ito and Yanofsky, 1969) and of anthranilate by L-tryptophan (Gonzalez and Somerville, 1986).

Reaction and enzyme constants

Parameter	Value	Uncertainty	Reference	In current model
Reversible	no	-	-	-
k_{cat}	6.6 1/s	-	Jackson and Yanofsky, 1974 (calculated from specific activity)	no
k_{cat}	4.4 1/s	0.2 (chosen)	Gonzalez and Somerville, 1986 (calculated from specific activity)	yes
k_{appt}^{max} x2	2.27 x2 = 4.54 1/s	0.2 (chosen)	Calculated from experimental fluxes and protein concentrations (ΔsdhCB)	no
Formation energy ANTH	-42.3 kJ/mol	5.7 kJ/mol	eQuilibrator	no
Formation energy PRPP	-2978.9 kJ/mol	3.3 kJ/mol	eQuilibrator	no
Formation energy PRAN	-1038.7 kJ/mol	7.5 kJ/mol	eQuilibrator	no
Formation energy PPi	-1960.7 kJ/mol	2.2 kJ/mol	eQuilibrator	no
K_M ANTH	0.00058 mM	0.2 (chosen)	Gonzalez and Somerville, 1986	yes
K_M PRPP	0.1 mM	-	Ito and Yanofsky, 1969	no
K_M PRPP	0.05 mM	0.2 (chosen)	Gonzalez and Somerville, 1986	yes
K_M PRAN	-	-	-	-
K_M PPi	-	-	-	-
K_M PRPP of trpD	0.2 mM	-	Ito and Yanofsky, 1969	no
K_I TRP	0.0005 mM	-	Pabst et al., 1973	no
K_I TRP	0.0005 mM	-	Gonzalez and Somerville, 1986	no

Tryptophan - PRAIi

Reaction equation

$1\ PRAN \rightarrow 1\ 2CPR5P$

N-(5-phosphoribosyl)anthranilate = 1-(2-carboxyphenylamino)-1-deoxyribulose 5-phosphate

BIGG: -1 pran_c +1 2cpr5p_c

Enzymes and genes

EcoCyc and UniProt

PRAI (phosphoribosylanthranilate isomerase) is a monomeric enzyme encoded by the *trpC* gene and is located in the cytosol. TrpC catalyses both the PRAI and IGPS reaction and mutant complementation studies demonstrated that the two reactions occur at two distinct, non-overlapping sites on the polypeptide and that 2CPR5P is a free intermediate (Creighton, 1970). The amino-terminal domain carries out the synthase activity and the carboxy-terminal domain carries out the isomerase activity (Eberhard and Kirschner, 1989), so channeling of the intermediate substrate is not likely. TrpC is unique among the five enzymes in the tryptophan biosynthesis pathway in that it is not part of a multisubunit enzyme complex (Christie and Platt, 1980). A Δ*trpC* mutant in not viable in minimal medium.

Regulation

Gene regulation: L-tryptophan binds to TrpR (DNA-binding transcription factor) to enable inhibition and TrpR inhibits transcription initiation of *trpC*. L-tryptophanyl (tRNA for L-tryptophan promotes premature termination of transcription.

Activators: None or unknown.

Inhibitors: None or unknown.

Reaction and enzyme constants

Parameter	Value	Uncertainty	Reference	In current model
Reversible	no	-	-	-
k_{cat}	38.9 1/s	0.2 (chosen)	Eberhard and Kirschner, 1989	yes
k_{cat}	40.0 1/s	-	Hommel et al., 1989	no
k_{cat}	40.0 1/s	-	Eberhard et al., 1995	no
Formation energy PRAN	-1038.7 kJ/mol	7.5 kJ/mol	eQuilibrator	no
Formation energy 2CPR5P	-1048.5 kJ/mol	7.1 kJ/mol	eQuilibrator	no
K_M PRAN	0.005 - 0.01 mM	-	Creighton, 1970	no
K_M PRAN	0.0071 mM	0.2 (chosen)	Eberhard and Kirschner, 1989	yes
K_M PRAN	0.0049 mM	-	Hommel et al., 1989	no
K_M PRAN	0.0049 mM	-	Eberhard et al., 1995	no
K_M 2CPR5P	-	-	-	-

Tryptophan - IGPS

Reaction equation

$1\ 2CPR5P \rightarrow 1\ 3IG3P + 1\ CO_2 + 1\ H_2O$

1-(2-carboxyphenylamino)-1-deoxyribulose 5-phosphate = (3-indolyl)-glycerol 3-phosphate + CO_2 + H_2O

BIGG: -1 2cpr5p_c +1 3ig3p_c (+1 co2_c +1 h2o_c)

Enzymes and genes

EcoCyc and UniProt

ISPS (indole-3-glycerol-phosphate synthase) is a monomeric enzyme encoded by the *trpC* gene and is located in the cytosol. TrpC catalyses both the PRAI and IGPS reaction and mutant complementation studies demonstrated that the two reactions occur at two distinct, non-overlapping sites on the polypeptide and that 2CPR5P is a free intermediate (Creighton, 1970). The amino-terminal domain carries out the synthase activity and the carboxy-terminal domain carries out the isomerase activity (Eberhard and Kirschner, 1989), so channeling of the intermediate substrate is not likely. TrpC is unique among the five enzymes in the tryptophan biosynthesis pathway in that it is not part of a multisubunit enzyme complex (Christie and Platt, 1980). A Δ*trpC* mutant in not viable in minimal medium.

Regulation

Gene regulation: L-tryptophan binds to TrpR (DNA-binding transcription factor) to enable inhibition and TrpR inhibits transcription initiation of *trpC*. L-tryptophanyl (tRNA for L-tryptophan promotes premature termination of transcription.

Activators: None or unknown.

Inhibitors: Inhibition by anthranilate and derivatives (Smith and Yanofsky, 1962) and by rCdRP (Priestle et al., 1987).

Reaction and enzyme constants

Parameter	Value	Uncertainty	Reference	In current model
Reversible	no	-	-	-
k_{cat}	7.2 1/s	0.2 (chosen)	Eberhard and Kirschner, 1989	yes
k_{cat}	3.6 1/s	-	Eberhard et al., 1995	no
k_{cat}	2.7 1/s	-	Darimont et al., 1998	no
Formation energy 2CPR5P	-1048.5 kJ/mol	7.1 kJ/mol	eQuilibrator	no
Formation energy 3IG3P	-584.0 kJ/mol	7.3 kJ/mol	eQuilibrator	no
Formation energy CO_2	-403.1 kJ/mol	5.7 kJ/mol	eQuilibrator	no
K_M 2CPR5P	0.005 mM	-	Creighton and Yanofsky, 1966	no
K_M 2CPR5P	0.0012 mM	0.2 (chosen)	Eberhard and Kirschner, 1989	yes
K_M 2CPR5P	0.00042 mM	-	Eberhard et al., 1995	no
K_M 2CPR5P	0.0003 mM	-	Darimont et al., 1998	no
K_M 3IG3P	-	-	-	-
K_M CO_2	-	-	-	-

Tryptophan - TRPS1

Reaction equation

$1\ 3IG3P + 1\ SER \rightarrow 1\ TRP + 1\ G3P + H_2O$

(3-indolyl)-glycerol 3-phosphate + L-serine = L-tryptophan + glyceraldehyde 3-phosphate + H_2O

BIGG: -1 3ig3p_c -1 ser_L_c +1 trp_L_c +1 g3p_c (+1 h2o_c)

Enzymes and genes

EcoCyc and UniProt trpA, UniProt trpB

The TRPS (tryptophan synthase) reaction is catalysed by the TrpAB complex, a heterotetrameric enzyme (two A subunits and one dimer of B) located in the cytosol and encoded by the *trpA* and *trpB* genes. The overall tryptophan synthase reaction consists of a sequence of two partial reactions. The α subunit of the complex carries out the aldol cleavage of 3IG3P to indole and G3P. The β subunit is responsible for the synthesis of L-tryptophan from indole and L-serine. The intermediate substrate (indole) is channeled through the enzyme complex and does not appear in solution. (Miles et al., 1999) The TrpA monomer is able to catalyse the first part of the reaction, however within the physiological complex with the B subunit, the reaction rate is increased by 1-2 orders of magnitude (Lim et al., 1991, Kirschner et al., 1991). The TrpB dimer is seems to be able to catalyse the second part of the reaction (Kaufmann et al., 1991). PLP (pyridoxal 5-phosphate) is a cofactor of the enzyme complex, because two PLP molecules bind to the B dimer. Both mutants are not viable on minimal medium.

Regulation

Gene regulation: L-tryptophan binds to TrpR (DNA-binding transcription factor) to enable inhibition and TrpR inhibits transcription initiation of *trpA* and *trpB*. L-tryptophanyl (tRNA for L-tryptophan promotes premature termination of transcription.

Activators: None or unknown.

Inhibitors: The partial reaction of the A subunit is competitively inhibited by indolepropanol phosphate (Kirschner et al., 1975).

Reaction and enzyme constants

Parameter	Value	Uncertainty	Reference	In current model
Reversible	no	-	(only A part is reversible)	-
k_{cat}	23.4 1/s	-	Jackson and Yanofsky, 1974 (calculated from specific activity)	no
k_{cat}	1.5 1/s	-	Kirschner et al., 1991	no
k_{cat}	3.8 1/s	-	Banik et al., 1995	no
k_{cat}	1.4 1/s	0.2 (chosen)	Lane and Kirschner, 1991	no
k_{cat}	4.7 1/s	0.2 (chosen)	Anderson et al., 1995	yes
k_{appt}^{max} x2		-	Calculated from experimental fluxes and protein concentrations (WT)	no
Formation energy 3IG3P	-584.0 kJ/mol	7.3 kJ/mol	eQuilibrator	no
Formation energy SER	-233.5 kJ/mol	2.2 kJ/mol	eQuilibrator	no
Formation energy TRP	-393.5 kJ/mol	4.2 kJ/mol	eQuilibrator	no
Formation energy G3P	-1091.5 kJ/mol	1.3 kJ/mol	eQuilibrator	no
K_M 3IG3P	0.03 mM	-	Kirschner et al., 1991	no
K_M 3IG3P	0.03 mM	-	Banik et al., 1995	no
K_M 3IG3P	0.069 mM	0.2 (chosen)	Lane and Kirschner, 1991	yes
K_M SER	14.5 mM	-	Crawford and Ito, 1964	no
K_M SER	0.34 mM	0.2 (chosen)	Lane and Kirschner, 1991	yes
K_M TRP	-	-	-	-
K_M G3P	0.7 mM	-	Kirschner et al., 1991	no
